# A network medicine approach to quantify distance between hereditary disease modules on the interactome

**DOI:** 10.1038/srep17658

**Published:** 2015-12-03

**Authors:** Horacio Caniza, Alfonso E. Romero, Alberto Paccanaro

**Affiliations:** 1 Department of Computer Science, Centre for Systems and Synthetic Biology, Royal Holloway, University of London, Egham Hill, Egham, UK

## Abstract

We introduce a MeSH-based method that accurately quantifies similarity between heritable diseases at molecular level. This method effectively brings together the existing information about diseases that is scattered across the vast corpus of biomedical literature. We prove that sets of MeSH terms provide a highly descriptive representation of heritable disease and that the structure of MeSH provides a natural way of combining individual MeSH vocabularies. We show that our measure can be used effectively in the prediction of candidate disease genes. We developed a web application to query more than 28.5 million relationships between 7,574 hereditary diseases (96% of OMIM) based on our similarity measure.

Over recent decades advances in proteomics have resulted in considerable gains in our understanding of heritable diseases and our perspective has evolved from simple gene-disease associations to considering diseases as perturbations in regions of the interactome – the disease modules^1^. In this context, related diseases are associated with close-by regions^2^,^3^. Quantifying disease similarity at molecular level would allow the transfer of knowledge between similar diseases^4^, possibly providing hypotheses for causal genes discovery and even suggestions for drug repositioning.

Few methods for quantifying disease similarity at molecular level have recently appeared (see Supplementary material § 6). The method proposed by Park *et al.*^5^ calculates similarity between diseases as an association score between the different disease proteins based on their subcellular co-localisation. van Driel *et al.*^4^ present a measure based on text-mining analysis of the disease phenotype descriptions found in the OMIM compendium of heritable diseases^6^. These descriptions are mined for a predefined set of Medical Subject Headings (MeSH) terms which are used to construct feature vectors for every disease. Similarity between diseases is then given by the cosine of the angle between their respective feature vectors. Zhou *et al.*^7^ extract diseases and symptoms from MeSH, and through the mining of PubMed metadata they construct feature vectors describing each disease in terms of its symptoms. Similarly to van Driel *et al.,* the similarity between two diseases is given by the cosine of the angle between their respective feature vectors, followed by a filtering of similarities based on statistical significance. Robinson *et al.*^8^ explore a different approach by manually constructing the Human Phenotype Ontology (HPO). This ontology provides a standardised vocabulary for phenotypic information which is used to annotate OMIM diseases. Similarity between diseases is calculated using an information content-based similarity measure on the HPO.

## Results

The approach we present here attempts to summarize existing information about diseases through large scale analysis of hand curated data. Our method is based on the idea that, for a given disease in OMIM, the set of MeSH terms annotating the publications referenced by its OMIM entry accurately describes that disease. This allows us to establish a mapping between diseases in OMIM and the MeSH ontology: every disease is annotated by the set of MeSH terms associated with its publications. Next, we use the structure of MeSH to measure the semantic similarity between the sets of terms annotating the diseases (see **Methods**). Importantly, terms in MeSH are organised into 16 ontologies according to thematic domains (e.g. Anatomy). Since a disease can be annotated by terms from several ontologies, this results in (up to) 16 similarity scores for each pair of diseases. Our in-depth analysis of MeSH revealed large overlaps between the ontologies (see **Methods** and Supplementary Discussion § 3 and 11) and we exploit this interconnectedness between the ontology structures in order to produce a single score which effectively encapsulates the diverse information available from the literature. In the following we show that our measure accurately reflects associations between underlying genes and proteins, hence characterising the relatedness between diseases at molecular level.

**Figure 1:**
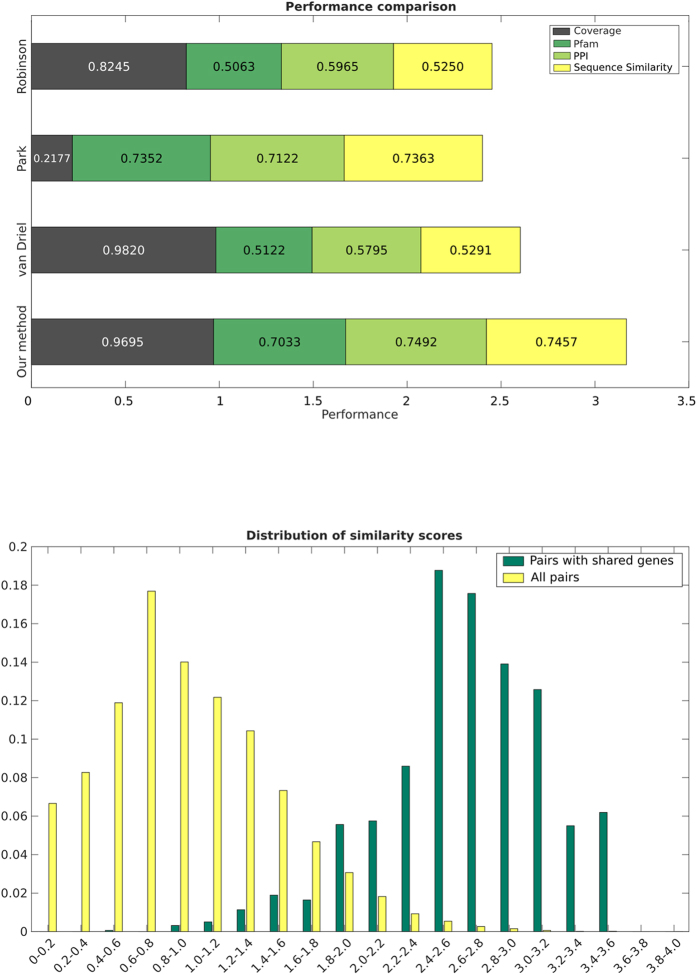
Top) Performance Comparison. For each method, the grey bar quantifies its OMIM coverage, coloured bars quantify its performance measured by AUCs on the Pfam, PPI and Sequence Similarity datasets. The total length of each bar represents the overall performance of each method. Bottom) Comparison of score distributions. Distribution of similarity scores for all pairs of diseases (yellow bars) vs. distribution of similarity scores for disease pairs sharing one or more disease genes (green bars). 90% of the pairs of diseases with shared genes have scores in the 99^th^ percentile or higher.

To evaluate our measure and compare it to previous ones, we follow the approach used by van Driel *et al.*^4^ who proposed to quantify the molecular level similarity between diseases using three relationships between their disease proteins, namely physical interactions, domain co-occurrence based on Pfam^9^ and sequence similarity (see **Methods** and Supplementary material § 8). Thus, the evaluation is reduced to a binary classification problem, where disease similarity scores are used to predict these binary relationships. The performance of the measure is evaluated by computing the area under the ROC curve (AUC). Finally, another important criterion for a disease similarity method to be of practical importance is its wide applicability. Therefore, in our evaluation we included coverage, defined as the percentage of OMIM diseases for which similarities can be computed (see Supplementary material § 4). Figure 1 **Top** presents a comparison between our method and a representative set of other approaches namely Park^5^, van Driel^4^ and Robinson^8^. Both larger AUC values and larger coverage are better, and since these scores are all bound between 0 and 1, we sum them into a composite score to compare the methods’ overall performance. The figure shows that our method outperforms earlier approaches. We also separately evaluated the performance of our measure on multigenic and monogenic diseases and we found it to be comparable (see Supplementary material § 19, 23).

To further assess the correlation of our similarity measure with the molecular level similarity, we contrast the distribution of similarity scores for all pairs of diseases with that of the subset of pairs sharing disease genes. This comparison is shown in Fig. 1 **Bottom** as normalised histograms. The two distributions are very different (Student’s *t*-test *P* < 10^−350^). 90% of the pairs of diseases with shared genes have high-similarity scores (99^th^ percentile or higher), indicating that high-similarity values are correlated with existing knowledge of relatedness at molecular level (see Supplementary material § 7, 12, 14, 21).

For many disease pairs with high similarity scores, we could readily verify that they are indeed similar at molecular level by analysing existing medical literature. For example, the score between Budd-Chiari (MIM: 600880) syndrome and Myeloproliferative disorder (MIM: 131440) is in the 97^th^ percentile and genes associated to these diseases have *in vivo* verified first-level interactions (JAK2 – PDGFRB). Furthermore, it is known that these two diseases are causally related^10^. The score between Breast Cancer (MIM: 114480) and Noninsulin Dependent Diabetes (NDDIM) (MIM: 125853) lies in the 100^th^ percentile, and several cancer related proteins are known to interact with NDDIM related proteins (TP53 – HNF4A, CDH1 – PTPN14, CDH1 – IRS1). Moreover, there exists statistical evidence of increased risk of Breast Cancer in Women with type 2 diabetes^11^. The similarity scores between Type I von Willebrand disease (VWD1) (MIM: 193400) and pseudo von Willebrand disease (VWDP) (MIM: 177820), two bleeding disorders, lies in the 100^th^ percentile. VWD1 is a consequence of exceptionally low levels of plasma von Willebrand Factor (VWF)^12^, while VWDP is characterised by subtle mutations in the alpha subunit of the glycoprotein Ib (GPIbα) subunit, causing it to bond uncharacteristically to VWF^13^.

One of the possible applications of our method lies in the transferring of knowledge between diseases and particularly in the prediction of candidate disease genes. To assess its effectiveness for this task, we built “old” similarity scores using an older version of OMIM (downloaded on April 9^th^, 2013) and found that several pairs of diseases which had high similarity values according to data from 2013, have since been shown to be close on the interactome. For example, our 2013 version of OMIM reports no disease genes for SHORT syndrome (MIM: 269880), Dermatofibrosarcoma protuberans (MIM: 607907) and Right Atrial Isomerism (MIM: 208530). However, our “old” similarity scores indicate SHORT syndrome to be very similar at molecular level to Noninsulin-dependent Diabetes Mellitus (MIM: 125853) (99th percentile), thus suggesting that disease genes for SHORT syndrome could be located in the neighbourhood of Diabetes. This is indeed the case, as the new version of OMIM links SHORT syndrome to gene PIK3R1, which has a verified *in-vitro* interaction with IRS1, a gene associated to noninsulin-dependent diabetes. Similarly, our “old” similarity scores indicate Dermatofibrosarcoma to be very similar at molecular level to Juvenile Myelomonocytic Leukemia (MIM: 607785) (100th percentile). The current version of OMIM shows an association between Leukemia and the gene PDGFRB, which interacts with PDGFB a gene associated to Dermatofibrosarcoma; the “old” score between Right Atrial Isomerism and Tetralogy of Fallot (MIM: 187500) is in the 100th percentile and now it has been shown that they share a disease gene (GDF1). The list of publications available in the 2013 version of OMIM for each of the above diseases can be found in the Supplementary Discussion § 18.

By exploring simpler measures based on the overlap between sets of MeSH terms, we prove that exploiting the structure of the MeSH ontology is essential to accurately quantify similarity between diseases at molecular level (see Supplementary Discussion § 9). Finally, we show that the high accuracy of our similarity measure is due to both the quality of the MeSH terms which our approach assigns to OMIM diseases, as well as to the way in which our method uses the ontology structure (see Supplementary Discussion § 9, 15 and 16).

**Figure 2:**
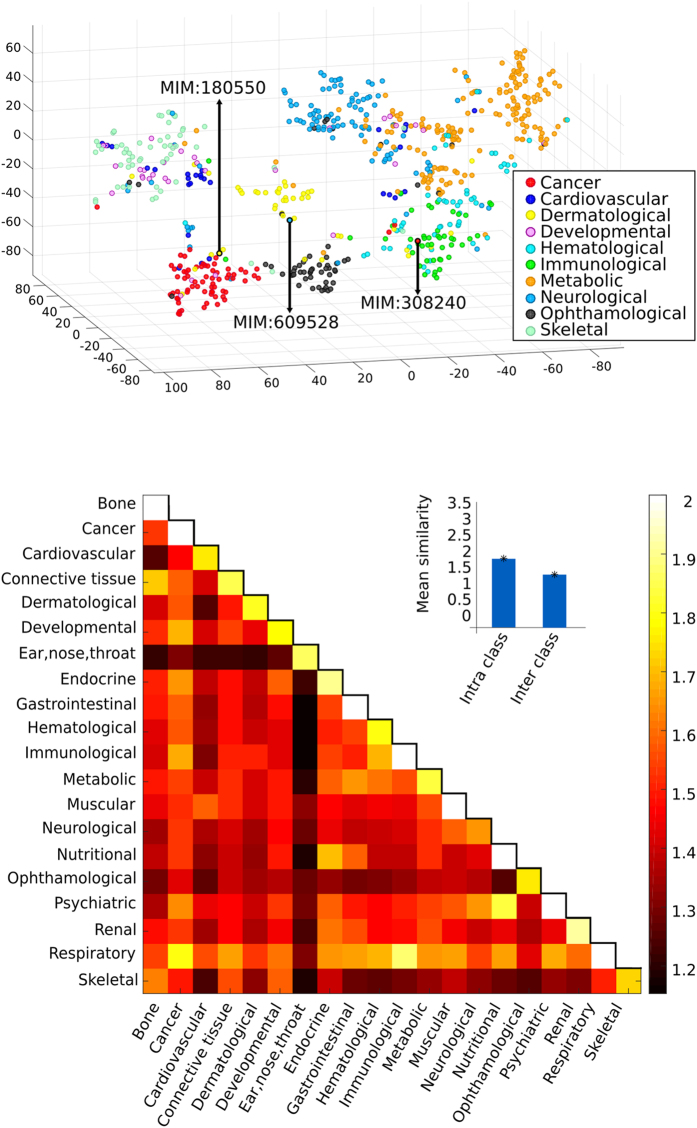
Top) Embedding of hereditary diseases in 3D space using t-SNE. Each point represents an OMIM disease. Colours are assigned based on their disorder class according to Goh *et al.*^3^. Highlighted diseases belong to multiple phenotypic classes and are discussed in the main text. Bottom) Heat map of mean class similarities. Each (x, y) tile represents, for the disease classes in Goh *et al.*^3^, the mean similarity of disease pairs where one disease belongs to class x and the other to class y. The values range from 1.15 (Gastrointestinal – Ear, nose, throat) to 2.71 (Nutritional-Nutritional). The colours range between the minimum mean similarity and 2, with all values above 2 (In the diagonal: 2.01 Bone, 2.05 Immunological, 2.06 Gastrointestinal, 2.07 Muscular, 2.1 Psychiatric, 2.2 Cancer, 2.5 Respiratory, 2.71 Nutritional) set to 2. Inset: the average intra-class similarity is significantly higher than the average inter-class similarity (t-test p-value < 10^−350).

Our measure allows us to obtain a 3D graphical representation of human diseases^3^ automatically. Figure 2 **Top** shows the embedding of diseases into 3D space obtained applying t-SNE^14^, a recently developed dimensionality reduction technique. In the figure, each point corresponds to a disease and the distance between two diseases relates to our similarity measure. Each disease is coloured according to the disease classes of Goh *et al.*^3^ who categorise each disease in OMIM into 19 classes according to the physiological system it affects. The categories are: Bone, Cancer, Cardiovascular, Connective tissue disorder, Dermatological, Developmental, Ear-Nose-Throat, Endocrine, Gastrointestinal, Haematological, Immunological, Metabolic, Multiple, Muscular, Neurological, Nutritional, Ophthalmological, Psychiatric, Renal, Respiratory and Skeletal. In the figure we show the diseases in the 10 most numerous classes (see Supplementary Discussion § 17). This plot reveals that diseases in the same class tend to be grouped together. This is interesting, as Goh *et al.* showed that these classes group diseases that are highly related at molecular level (see Supplementary Discussion § 17).

Notice how some diseases which, from a phenotypical perspective belong to multiple classes, are placed appropriately at the boundaries between them (see diseases pointed by arrows in Fig. 2 **Top**). For example the Ring dermoid of Cornea (MIM: 180550), is located at the boundary between the Dermatological, Cancer and Ophthalmological classes. This disease is characterised by dermoids (growths with a skin-like structure) in the eye; dermoids, in general, exhibit known hallmarks of cancer^15^. Cerebral dysgenesis, neuropathy, ichthyosis, and palmoplantar keratoderma syndrome (MIM: 609528) is characterised by severe neurological impairment as well as keratoderma and late-onset ichthyosis^16^. Our embedding places this disease at the boundary between the Neurological and Dermatological classes. In other cases, diseases that belong to more than one class are placed closer to a class different from the one chosen by Goh *et al.*^3^, but their position is overall appropriate when considering the diseases’ characteristics. For example, lymphoproliferative syndrome, X-linked, 1 (MIM: 308240), exhibits both immunological and cancer features. It is characterised by severe immunological dysregulation, and is related to several phenotypes (including lymphoma) and often occurs after an infection (Epstein-Barr virus). Our embedding places this disease closer to immunological diseases than to the cancer group. We discuss boundary diseases in more detail in Supplementary Discussion § 20.

The clear grouping of diseases is made possible by the difference between average inter- and intra-class similarity values – these are visualised as a heat map in Fig. 2 **Bottom**. We also note that pairs of classes with high average inter-class similarity contain diseases which are often related. For example, this can be the case for diseases in the immune and respiratory classes as it is known that an abnormal immune response can cause chronic respiratory diseases^17^.

We provide a full interactive browser at http://www.paccanarolab.org/disimweb which enables the user to obtain the similarity measure between over 28.5 million pairs of diseases. Connections to OMIM, MeSH and UniProtKB databases are also provided. The data and source code used to generate the similarity scores as well as the website is available for download from the same website.

We have also developed the Disease Similarity Resource (DSR), a database of disease pairs whose similarity is in the top 5%. Each pair of diseases defines an entry with 5 columns: Disease A, Disease B, Similarity score, UniProt/KB identifiers of the proteins associated to disease A followed by those associated to disease B. These 1,552,356 pairs of highly similar diseases are a starting point for the analysis of the relationships between diseases as well as for the discovery of new disease genes (see Supplementary material § 22). The DSR is available from http://www.paccanarolab.org/disimweb in the “Download” section.

## Discussion

In this paper we have introduced a method to obtain a high-quality score that characterise disease similarity at molecular level. We have shown that our method can be used to predict diseases whose modules are located close on the interactome, allowing the transfer of knowledge between them. We can envision an interactive differential diagnosis system that would aid medical practitioners in identifying putative alternative diagnoses that are obscured by the complexity and multiplicity of the symptoms.

Our method annotates diseases using the MeSH terms associated to the publications found in OMIM and then combines these annotations with the structure of the MeSH ontology. One important question is whether the method’s performance is due to the quality of the annotations, or to the way in which it exploits the structure of the MeSH ontology, or to both.

**Figure 3:**
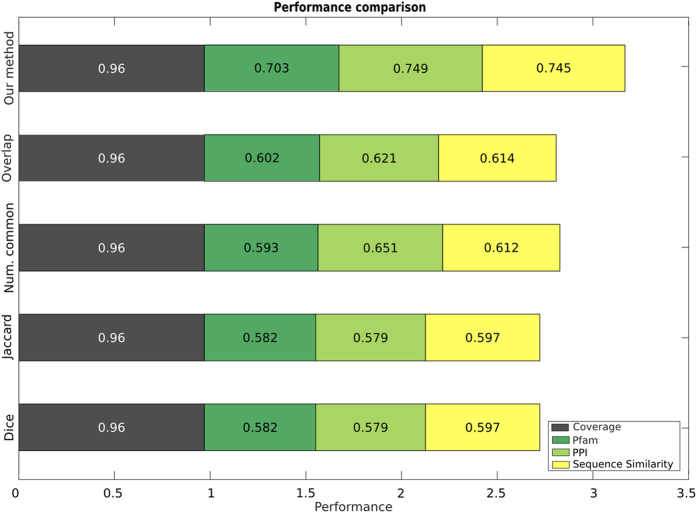
Effects of MeSH’s ontological structure. The performance of our method, which uses the MeSH ontology structure, is better than the simpler, overlap based methods. For each method, the grey bar quantifies its OMIM coverage, coloured bars quantify its performance measured by AUCs on the Pfam, PPI and Sequence Similarity datasets. The total length of each bar represents the overall performance of the method.

**Figure 4:**
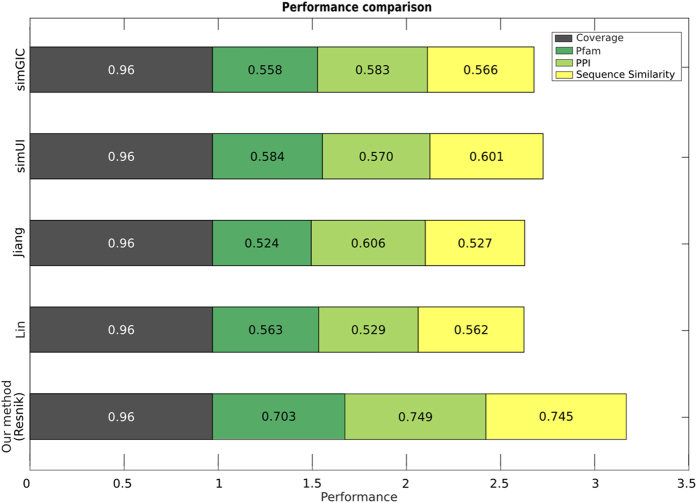
Correct use of the ontology structure. The improved performance of Resnik’s measure, used by our method, is due to a better use of the ontological structure. For each method, the grey bar quantifies its OMIM coverage, coloured bars quantify its performance measured by AUCs on the Pfam, PPI and Sequence Similarity datasets. The total length of each bar represents the overall performance of the method.

In order to quantify the effects of MeSH’s ontological structure, we analysed the performance of similarity measures which disregard the ontology structure and are simply based on calculating the overlap of the MeSH terms annotating the diseases (See **Methods**). Therefore, these measures produce scores that do not depend on the specificity of the MeSH terms, equally weighing specific terms (*e.g.* Metatarsus - D008684) and broad ones (*e.g.* Body Regions - D001829). Figure 3 presents a comparison between our method and these simple measures on the Pfam, PPI and Sequence Similarity datasets. While the coverage is the same as for our method, the performance of these simpler measures is inferior. When looking at the ROC curves in detail (see Supplementary figures 3b–3d) we understand that, as expected, these measures are conservative, being able to correctly produce high scores for very similar diseases, but being unable to provide appropriate lower scores for pairs of less similar diseases. It is important to note, however, that while the use of MeSH’s ontological structure improves performance significantly not all semantic similarity measures are well suited for the MeSH ontology. A comparison with the semantic similarity measures by Lin^18^, Jiang^19^, simUI^20^ and simGIC^20^ (see **Methods**) shows that the measure by Resnik, used in our method, performs best (see Fig. 4 and Supplementary Discussion § 10). The lower performance of Lin’s and Jiang’s methods is due to the fact that using these measures, if the sets of MeSH terms annotating two diseases overlap, their similarity will always be maximal, irrespective of the specificity of the terms in the annotations. This is not a problem when calculating semantic similarities between genes using the Gene Ontology, as gene GO annotations in general overlap little compared to disease Mesh annotations—see Supplementary Figure 5 which compares the overlap of MeSH terms for OMIM diseases with the overlap of GO terms for genes in *A. thaliana, H. sapiens, M. musculus, S. cerevisiae* and *C. elegans*. Therefore, Lin’s and Jiang’s measures produce an incorrectly large proportion of high-similarity pairs. Conversely, simUI and simGIC, although they exploit the structure of the ontology to expand the set of terms, are ultimately based on the overlap of MeSH terms and therefore behave similarly to the aforementioned simpler measures.

In order to quantify the effects of the quality of our disease annotations, we replaced them with the OMIM-to-MeSH mapping used by van Driel’s *et al.* This was possible due to the fact that van Driel also uses sets of MeSH terms to annotate the diseases. However, these annotations were obtained text-mining the Clinical Synopsis and Text fields of OMIM for terms in the A (Anatomy) and C (Diseases) ontologies in MeSH. Supplementary figures 29, 30 and 31 show the evaluation results for the Pfam, PPI and Sequence Similarity datasets highlighting the fact that the sets of MeSH terms associated with the publications for a given disease are informative descriptors for that disease.

Our analysis shows that the MeSH terms associated to the publications referenced in OMIM are good descriptors of the diseases themselves, and that the MeSH ontology structure provides valuable information for calculating distances between sets of terms. Combining these two, we obtain a high-quality score that characterises disease similarity at molecular level.

## Methods

OMIM entries describe individual diseases and are composed of several plain text fields as well as references to scientific publications provided in the form of PubMed identifiers. These identifiers provide access to the MEDLINE entry for the linked reference from which metadata can be retrieved, including the MeSH terms. From the 21st of July 2014 version of OMIM we obtained 7,574 disease phenotypes referencing 62,830 publications. These publications were associated with 13,220 MeSH Main Heading terms which we used to annotate the OMIM diseases. The “old” OMIM dataset, used to showcase the potential of our method to predict disease genes, corresponds to the release of April 9^th^ 2013 of OMIM. This version contains 7,525 diseases referencing 61,889 publications annotated with 13,006 MeSH Main Heading terms. For details, please refer to Supplementary material § 2 and 11.

Experimental results presented here use the similarity measure proposed by Resnik^21^. Resnik’s semantic similarity between two terms in an ontology is based on the concept of information content of a term, defined as the negative logarithm of the probability of that term (calculated as the ratio between the number of diseases annotated by that term and the total number of annotated diseases). The similarity of two terms 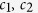
is defined as the information content of their common ancestor with highest information content, that is:


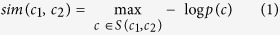


where 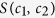 is the set of common ancestors of 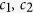 and 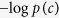 is the information content of term 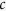. We defined the similarity of two diseases 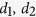 as the maximum similarity for all possible pairs of MeSH terms 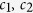 annotating the disease pair, that is:


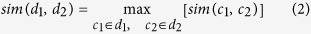


**Figure 5:**
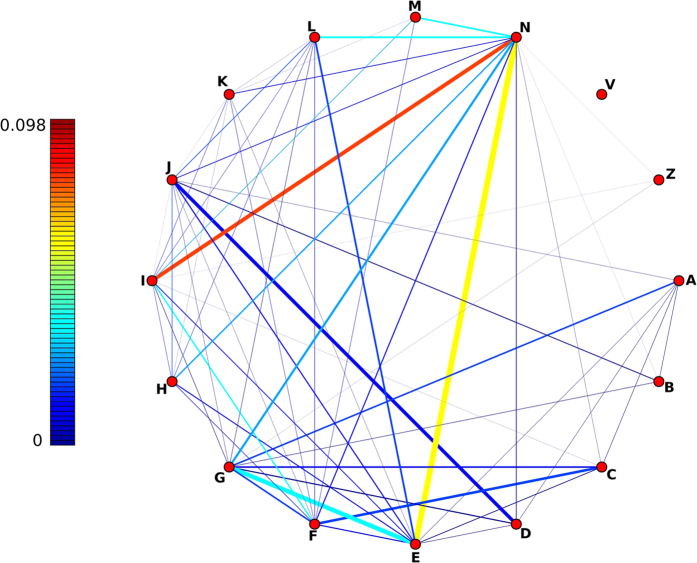
Overlap of the MeSH ontologies. Nodes represent MeSH ontologies and links are related to the amount of overlap between them. Link colours correspond to the Jaccard coefficient between the set of terms in each pair of ontologies. Link thicknesses correspond to the number of shared terms between ontologies and only strictly positive links are shown. MeSH Ontologies abbreviations: [A] Anatomy, [B] Organisms, [C]Diseases, [D] Chemicals and drugs, [E] Analytical, Diagnostic and Therapeutic Techniques and Equipment, [F] Psychiatry and Psychology, [G] Phenomena and Processes, [H] Disciplines and Occupations, [I] Anthropology, Education, Sociology and Social Phenomena, [J] Technology, Industry, Agriculture, [K] Humanities, [L], Information Science, [M] Named Groups, [N] Health Care, [V] Publication Characteristics, [Z] Geographical.

Thus, for every pair of diseases in OMIM, we obtain a different similarity score for each MeSH ontology in which both diseases are annotated. Our analysis of the interconnectedness of the MeSH ontologies allowed us to combine them, thus obtaining a single similarity score for each pair of diseases. The basis for the combination lies in the fact that some terms are shared between MeSH ontologies, and this overlap creates a series of paths which link them together into a single ontological structure. Figure 5 shows the pairwise overlap between the different ontologies quantified by their Jaccard coefficient. Results presented here are obtained using the ontologies which had an AUC above 60% for the PPI dataset while maintaining a high coverage of OMIM diseases, namely Anatomy [A] (6,781 diseases), Diseases [C] (7,321 diseases), Chemicals and Drugs [D] (7,575 diseases), Analytical, Diagnostic and Therapeutic Techniques and Equipment [E] (7,000 diseases) and Phenomena and Processes [G] (7,018 diseases). We also tried other combinations and we found results to be equivalent as long as we included ontologies with high coverage. Performance and coverage of the proposed method in each individual ontology is shown in the Supplementary Discussion (**§ 12, 13 and 14**). As for most measures of semantic similarity between genes, our disease similarity measure is an unbounded, non-negative real number. We chose not to apply any order preserving transformation in order to rescale the scores, as it would have no effect on performance and could make it dataset-dependent or lead to misinterpretations (*e.g.* values constrained between zero and one might be wrongly interpreted as probabilities).

For the evaluation of our disease similarity measure and its comparison with existing measures, we follow the approach presented by van Driel *et al.*^4^, and assess the accuracy of our scores with respect to three binary relationships defining molecular relatedness between the 4,030 diseases with known proteins. The different measures are evaluated by comparing their performance on a classification problem, where the disease similarity scores are used to predict molecular level similarity, as represented by the three relationships. The relative performance of the different measures can then be compared using the Area under the ROC curve (AUC)^22^. We acknowledge that this evaluation is far from perfect due to limitations in the available molecular information of the diseases.

The first relationship proposed by van Driel *et al.* determines molecular relatedness based on protein-protein interactions between disease proteins. Two diseases are related if any of their disease proteins interact according to the Human Protein Reference Database (HPRD). This relationship resulted in 15,515 disease pairs relating 2,512 OMIM diseases. The second relationship is based on the co-occurrence of Pfam-A signatures (*i.e.* families, domains, motifs or repeats), and it relates two diseases if any of their disease-proteins share at least one of these signatures. After excluding disease pairs in which Pfam-A signatures associated to proteins in the pair matched a MeSH term as well as disease pairs with identical proteins, this relationship results in 33,660 pairs relating 2,647 OMIM diseases. The last relationship proposed by van Driel *et al.* is based on sequence similarity, and it relates two diseases whenever any of their disease proteins are similar in sequence. Sequence similarity is determined with a Smith-Waterman local alignment of the sequences with a threshold e-value smaller or equal to 10^-6. After excluding disease pairs with identical proteins this criterion results in 37,486 diseases pairs relating 2,817 OMIM diseases. Further details on the construction of these test datasets can be found in the Supplementary Discussion § 8.

The visualisation presented in Fig. 2 **Top** results from a 3D embedding of the diseases using t-SNE^14^ using the default parameters (perplexity set to 30 and number of dimensions for PCA pre-processing set to 50). The figure shows the diseases in the 10 most populated classes of Goh *et al.* (661 diseases in total).

We compared the performance of our measure with that of four simpler similarity measures (Jaccard, Dice, Overlap, Num. Common) which are based on calculating the overlap of the MeSH terms annotating the diseases and do not exploit the MeSH ontological structure. Given two diseases, *a* and *b*, their similarity *sim*_*(a, b)*_, is defined as follows:

1. Jaccard: uses the Jaccard coefficient of their respective annotation sets. Formally:


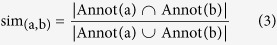


2. Dice: uses the Sørensen–Dice coefficient of their respective annotation sets. Formally:


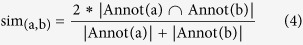


3. Overlap:


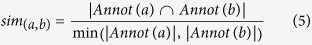


4. Num. Common: the size of the intersection of their annotation sets. Formally:


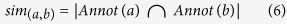


We compared the performance of our method which uses the Resnik measure, with that of four alternative similarity measures (Lin, Jiang, simUI, simGIC), which also exploit the ontology structure, by considering all the terms in the path to the root (True Path Rule).

1. Lin^18^: uses the normalised Resnik’s measure to account for the divergence between the terms:


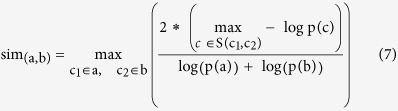


2. *Jiang*^19^: uses a distance measure:


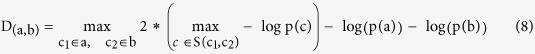


This distance measure is then transformed into a similarity score:


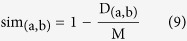


where *M* is the maximum possible value of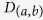.

3. *simUI*^20^:


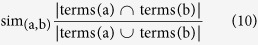


4. *simGIC*^20^: improves on simUI and it is based on a weighted Jaccard index, where the weight of each element is its information content^22^. Similarity between two diseases *a, b* is defined as:


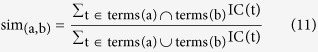


Additional details of these alternative similarity measures can be found in the supplementary material (see Supplementary Discussion § 5).

## Additional Information

**How to cite this article**: Caniza, H. *et al.* A network medicine approach to quantify distance between hereditary disease modules on the interactome. *Sci. Rep.* **5**, 17658; doi: 10.1038/srep17658 (2015).

## Supplementary Material

Supplementary Information
