# Supplementary material for: A network medicine approach to quantify distance between hereditary disease modules on the interactome
Source: Sci Rep. 2015 Dec 3;5:17658. doi: 10.1038/srep17658 (PMC4668371; doi:10.1038/srep17658)
Supplement: Supplementary Information [file srep17658-s1.pdf]

Supplementary Material to:

A network medicine approach to quantify distance between hereditary disease modules on the interactome

Horacio Caniza[1], Alfonso E. Romero[1] and Alberto Paccanaro[1]

[1]Department of Computer Science, Centre for Systems and Synthetic Biology, Royal Holloway, University of London, Egham Hill, Egham, UK.

Correspondence should be addressed to A.P. (alberto@cs.rhul.ac.uk)

1. Contents

1. Contents 2

2. A brief introduction to The Online Mendelian Inheritance in Man 4

OMIM and the links to MEDLINE 4

OMIM shows an imbalance in the study of inherited diseases 4

3. A brief introduction to *The Medical Subject Headings* thesauri 5

4. Exploring the limiting factors for our coverage of the OMIM diseases 6

5. A brief description of the semantic similarity measures analysed 7

Resnik [4] 8

Jiang and Conrath [6] 9

simUI [7] 9

simGIC [7] 9

6. The disease similarity methods discussed in the paper 9

van Driel. 9

Zhou. 10

Park *et al.* 10

Robinson *et al.* 10

Simple similarity measures 10

7. Mapping the existing disease similarity measures to OMIM 11

8. Details on the construction of the evaluation datasets. 12

Pfam dataset 12

PPI dataset 14

Sequence similarity dataset 15

9. Using the MeSH ontological structure improves the accuracy of disease similarity calculations 15

10. Correct use of the MeSH ontological structure is essential for accurate disease similarity calculations 18

11. The choice of MeSH term sets: All terms vs. Major topics 20

12. Performance of the measure in the individual ontologies 21

13. Combining the MeSH ontologies. Performance plots. 28

14. Performance comparison of existing methods of disease similarity. ROC plots and Bar charts 31

15. Ontological similarities in MeSH: A Two-step process 34

16. Small variability in scores for highly similar diseases 36

17. Details on the extracting the Human Disease Network classes 38

18. Details on the use of old versions of OMIM 38

19. Analysis of the performance for Complex diseases in OMIM 40

20. The boundary between Goh *et.al* disease classes. 42

21. The impact on the number of genes in a disease pair on its disease similarity score 44

22. The disease similarity resource. 47

23. Appendix: how to run our disease similarity pipeline 48

    Extracting the OMIM data: 48

    Extracting the referenced publications: 49

    Fetching the MeSH terms 49

    Producing the initial annotations 50

    Computing the matrices: 51

    Producing the benchmarks 51

24. References 54

2. A brief introduction to The Online Mendelian Inheritance in Man

OMIM [1] is a compendium of human phenotypes and their associated genes, focusing on the genotype-phenotype relation of all the known Mendelian disorders. Each entry consists of several free-text fields describing the phenotype as well as links to other resources. The entries are referenced with the relevant literature, through their PubMed identifiers. For the results presented in this paper we used the version of OMIM downloaded on 21st of July 2014.

OMIM records are prefixed with a character denoting its type (i.e. whether the entry describes a phenotype or a gene) and diseases are represented by four prefixes: "+", "#", "%" and "null", where "null" represents the lack of prefix. A total of 23,611 records comprise the entirety of OMIM, of which 7,812 correspond exclusively to diseases.

OMIM and the links to MEDLINE

Each OMIM record contains the hand-curated key references that describe the disease. OMIM entries do not provide "new" information, in fact, they are compendiums of the available knowledge in the literature and as such, the records are continually refined to reflect the latest knowledge available for a particular disease. A vast majority of references are specified in the form of PubMed identifiers.

We retrieve the PubMed identifiers for the OMIM diseases by querying the API, which results in 7,609 records mapped to 71,083 references, of which 62,829 are unique references. The 203 missing OMIM diseases correspond to entries for which no publication could be obtained through API queries to OMIM.

OMIM shows an imbalance in the study of inherited diseases

Figure 1 shows the number of publications the OMIM entries reference, reflecting the fact that highly-prevalent and easily diagnosed Mendelian disorders were elucidated first, leaving a large number of rare diseases understudied [2].

The majority of diseases (76%) references fewer than 10 publications and 99% of the OMIM records references fewer than 100 publications. The best referenced record is *MIM: 141900 - METHEMOGLOBINEMIA, BETA-GLOBIN TYPE, INCLUDED* with 1,094 publications. The next record, *MIM: 141800 - METHEMOGLOBINEMIA, ALPHA-GLOBIN TYPE, INCLUDED* follows with 387.

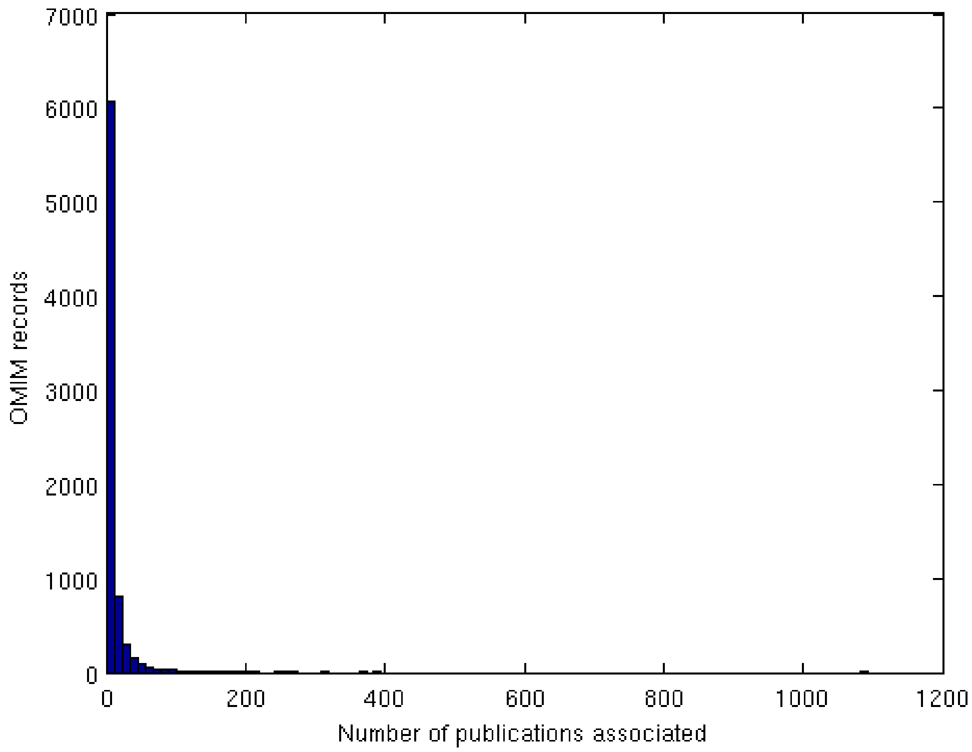

Figure 1 Number of referenced publications. This figure shows the number of OMIM entries (Y-axis) that reference a specific number of publications (X-axis).

3. A brief introduction to *The Medical Subject Headings* thesauri

The Medical Subject Headings (MeSH) is a controlled vocabulary designed to index biomedical literature in PubMed. MeSH is organised into 16 interconnected hierarchically organised ontologies describing different areas of knowledge. For example: *Respiratory System [A04]* is the hypernym of *Lung [A04.623],* and conversely, *Lung [A04.623]* is a hyponym of Respiratory *System [A04]*. In MeSH, each of the 16 ontologies is a Directed Acyclic Graph (DAG) and every descriptor can belong to more than one DAG.

Terms in MeSH are manually assigned to the publications in PubMed where they are used as indices for the publications. These terms are the relevant descriptors of the content of the publications. The following table presents the topological characteristics corresponding to the 2014 version of MeSH.

Table 1. MeSH ontologies

**Ontology**	**Terms**	**Max. depth**	**Avg. depth**	**Median depth**
[A] Anatomy	2,927	10	3.73	3
[B] Organisms	5,196	11	5.21	5
[C]Diseases	11,303	9	3.58	3
[D] Chemicals and drugs	20,992	10	4.53	4
[E] Analytical, Diagnostic and Therapeutic Techniques and Equipment	4,764	9	3.23	3
[F] Psychiatry and Psychology	1,150	6	2.96	3
[G] Phenomena and Processes	3,428	9	3.65	3
[H] Disciplines and Occupations	513	6	2.97	3
[I] Anthropology, Education, Sociology and Social Phenomena	651	8	3.52	3
[J] Technology, Industry, Agriculture	601	9	3.53	3
[K] Humanities	218	6	2.88	3
[L], Information Science	519	8	3.63	4
[M] Named Groups	258	6	2.68	3
[N] Health Care	2,350	8	3.70	4
[V] Publication Characteristics	188	3	1.34	1
[Z] Geographical	553	6	3.36	3

In the 2014 version of MeSH there are 27,149 terms, of which 13,220 are associated to the 62,829 publications referenced by OMIM diseases. The remaining 436 publications lacked, at the time of querying, PubMed MeSH terms.

4. Exploring the limiting factors for our coverage of the OMIM diseases

Our method covers 7,575 OMIM diseases corresponding to 96.9% of the total. The limiting factor for the coverage is the lack of MeSH annotations for some OMIM diseases. This can

occur in two different scenarios, either we are unable to retrieve the publication or the publication has no associated MeSH terms.

In the first case, there are two possibilities. The first possibility is that the OMIM disease does not reference any publication, such as the case of *Fragile Site 20p11* (MIM: 136590). The second possibility (this is the most common one), is that the publications that are referenced, are not indexed in PubMed. A total of 203 OMIM diseases fall in these two categories.

The second case relates to the lack of MeSH terms associated to few publications in PubMed. Out of the 62,829 PubMed identifiers available from OMIM, 62,393 are annotated with at least one MeSH term. The lack of annotations in the remaining publications occurs in the cases of newer publications which, at the time the publications were accessed, had not MeSH terms assigned to them. For example "*Human CalDAG-GEFI gene (RASGRP2) mutation affects platelet function and causes severe bleeding*" (PubMed ID 24958846) published in 2014. There were also a few PubMed entries without any MeSH terms annotating them without an evident reason, such as "*Some possible effects of nursing on the mammary gland tumor incidence in mice*" (PubMed ID 17793252), published in 1936, as well as more recent ones, such as "*Neonatal Hyperinsulinism*" (PubMed ID 10322395) published in 1999.

In some exceptional cases, the PubMed identifier referenced in OMIM entry referenced a non-existent PubMed identifier, such as in the case of MIM: 601419 referencing PubMed identifier 10553984. These cases were reported to the staff at OMIM.

5. A brief description of the semantic similarity measures analysed

Semantic similarity measures can be classified into term-based and graph-based [3]. Term-based measures determine similarities between pairs of terms in an ontology, while graph-based measures determine similarity between the annotated objects. We chose a representative set of each type of measure composed of some of the best known semantic similarity measures. For the term-based measures we chose Resnik [4], Lin [5], Jiang and Conrath [6] and for the graph-based measures simUI and simGIC [7].

Except for simUI, the aforementioned measures rely on the concept of information content of the ontology terms. The information content of the term c is defined as $IC(c) = -\log(p(c))$ where $p(c)$ is the probability of the term defined as the quotient between the

number of objects annotated with term c and the total number of objects annotated by the all other ontology terms.

The true-path rule states that an object annotated with a term c is also annotated by all ancestors of c. This results in $p(c)$ always being smaller or equal than the probability of any of its ancestors. Therefore, the information content decreases as one moves up the ontology, with the root having no information at all. That is, $IC(root) = 0$ implying that the probability of a random object being annotated to the root is 1, in accordance to the true-path rule.

For term based measures, the similarity of each pair of objects is determined by a matrix composed of the pairwise similarities between the terms annotating each object. If we consider a simple example with two objects annotated as follows $D_a = \{\mathrm{t1}, \mathrm{t2}\}$ and$D_b = \{t1\}$, the similarities between $\{\mathrm{t1}, \mathrm{t2}\}$ and $\{\mathrm{t1}, \mathrm{t1}\}$ should fully determine the similarity of D_a and D_b. To obtain a single similarity score for D_a and D_b a choice among the similarity of the individual pairs has to be made. A few options are available [3], such as choosing the similarity of the most similar pair, the similarity closest to the arithmetic mean or the median similarity. For this work we chose the similarity of the most similar pair.

Term base measures:

Resnik [4]

Resnik defines semantic similarity between two terms *a* and *b* in an ontology as the information content of the most informative ancestor, *i.e.* the lowest common ancestor *LCA (a, b)*:

$$sim(a,b) = -\log(P(LCA(a,b)))$$

Lin [5]

Lin's similarity measure normalises Resnik's proposal to account for the divergence between the terms:

$$sim_{LIN} = \frac{2 * \log(P(LCA(a,b))}{\log(P(a)) + \log(P(b))}$$

Jiang and Conrath [6]

Jiang and Conrath propose a distance measure:

$$D_{jiang} = 2 * \log(P(LCA(a,b)) - \log(P(a)) - \log(P(b))$$

Converting this distance into a similarity yields $sim_{jiang} = 1 - \frac{D_{jiang}}{M}$ where M is the maximum possible value of D_{jiang}.

Graph based measures

simUI [7]

simUI is based on the overlap of terms annotating two objects (o, p)

$$simUI(o,p) = \frac{|terms(o) \cap terms(p)|}{|terms(o) \cup terms(p)|}$$

simGIC [7]

simGIC is an improvement on simUI and it is based on a weighted Jaccard index, where the weight of each element is its information content [3]. Similarity between any two objects is defined as

$$simGIC_{(o,p)} = \frac{\sum_{t \in terms(o) \cap terms(p)} IC(t)}{\sum_{t \in terms(o) \cup terms(p)} IC(t)}$$

6. The disease similarity methods discussed in the paper

We begin this section by briefly describing the 4 disease similarity measures we discussed in the paper: the seminal work of van Driel *et al.* [8] and the methods by Park *et al.* [9], Robinson *et al.* [10] and Zhou *et al.* [11] which we refer to as van Driel, Park, Robinson and Zhou respectively.

At the end of this section we also present 4 simple similarity measures. Although these measures use MeSH terms to calculate disease similarities, they do so without exploiting the MeSH ontological structure. We have developed them to compare their results with our method, and thus prove that exploiting the MeSH ontological structure improves the results.

van Driel.

This approach is based on a text-mining analysis of OMIM, whereby diseases are classified based on features of their clinical descriptions. In their work, van Driel *et al.* use a subset of

MeSH (Anatomy (A) and Disease (C)) to select appropriate features which are used to produce a feature vector for each disease in OMIM. By automatically parsing the *Clinical Synopsis* field in the OMIM entries the selected features are tallied, fully defining the feature vector. The cosine of the angle of the vectors determines similarity between the diseases they describe.

Zhou.

Zhou *et al.* mine PubMed, extracting the MeSH terms associated to each publication and analyse the co-occurrence of a symptom term (terms in the C23 ontology in MeSH) and a disease term (terms in the C01-C26, except for the C22 and C23 ontologies in MeSH). This co-occurrence is compiled into a feature vector that characterises each disease based the frequency of its symptoms across PubMed. Similarity between every disease is obtained by computing the cosine of the angle between the feature vectors and then conserving only those statistically significant scores.

Park *et al.*

The main premise of Park's *et al.* work is that diseases whose proteins share a common subcellular localisation are phenotypically related. Similarity between two diseases is determined by an association score between diseases based on the cellular co-localisation of their disease proteins.

Robinson *et al.*

Robinson *et al.* propose the Human Phenotype Ontology (HPO), purpose-specific ontology. This manually curated ontology contains terms for the description of all phenotypic abnormalities for the diseases in OMIM. A text-mining analysis of OMIM, followed by careful manual curation, produces over 10,000 terms in the HPO. The ontology is then used to compute the semantic similarity between the diseases with an information-content based similarity measure.

Simple similarity measures

To keep this section self-contained we reproduce the definitions of the simple similarity measures presented in the **Methods** section of the main paper.

We constructed several simple disease similarity measures to explore the impact of the MeSH ontology structure on the accuracy of disease similarity measures.

The 4 simple similarity measures are:

1. Jaccard: The similarity of two diseases a, b is equivalent to the Jaccard coefficient of their respective annotation sets. Formally, $sim_{(a,b)} = \frac{|Annot(a) \cap Annot(b)|}{|Annot(a) \cup Annot(b)|}$
2. Dice: The similarity of two disease a, b is equivalent to the Sørensen–Dice coefficient of their respective annotation sets. Formally, $sim_{(a,b)} = \frac{2 * |Annot(a) \cap Annot(b)|}{|Annot(a)| + |Annot(b)|}$
3. Overlap: The similarity of two diseases a, b is given by $sim_{(a,b)} = \frac{|Annot(a) \cap Annot(b)|}{\min(|Annot(a)|, |Annot(b)|)}$
4. Number of common elements: The similarity of two diseases a, b is given by the size of the intersection of their annotation sets. Formally: $sim_{(a,b)} = |Annot(a) \cap Annot(b)|$

These similarity measures do not consider the ontological structure, and as such do not follow the true-path rule. Note that the Jaccard measure becomes the simUI measure when we follow the true-path rule.

7. Mapping the existing disease similarity measures to OMIM

We are able to produce comparisons with all the proposed measures, however in some cases a mapping is required due the fact that some disease similarity measures do not explicitly provide pairwise similarities between OMIM diseases.

In the case of Park *et al.* [9] the similarity scores are quantified between the syndromes defined by Goh *et al.* [12]. Park *et al.* use 1,284 of these syndromes from which we extracted a total of 1,717 OMIM diseases using the mapping detailed in Section 14 of this Supplementary material. Since a syndrome can group more than one OMIM disease, we replicated the same feature vector provided by Park for all OMIMs in the syndrome. The final similarity matrix was computed for all pairs of OMIM diseases mapped.

Zhou's *et al.* [11] HSDN (Human Symptoms Disease Network) compute similarity between diseases listed in the *Diseases [C]* ontology in MeSH. We first attempted to map the MeSH terms to the OMIM diseases by approximately matching the MeSH disease names and OMIM disease names calculating the Levenshtein distance, and positive matches were considered for strings with a matching ratio > 90%. This approach proved to be unsuccessful with an excessive number of false mappings. In a second attempt, we were able to match the MeSH disease terms to the OMIM diseases through the Disease Ontology (DO) [13].

Several DO entries contain a cross-reference field, which matches the DO term with corresponding entities in alternative databases. MeSH terms were matched to OMIM diseases based on co-occurrence in the same cross reference field in the DO. Unfortunately, this mapping had a very low coverage, resulting in only 454 OMIM diseases. Considering the poor coverage we cannot present an accurate representation of the performance of this measure.

8. Details on the construction of the evaluation datasets.

To ensure this section is self-contained, the following paragraph presents a brief synopsis of the evaluation criteria presented in the **Online Methods** of the paper.

For the evaluation of our diseases similarity measure we follow the approach presented by van Driel *et al.* [8], and assess the accuracy of our scores with respect to three binary relationships defining molecular relatedness between pairs of diseases.

Pfam dataset

The first relationship proposed by van Driel et al. is based on the co-occurrence of Pfam-A signatures (i.e. families, domains, motifs or repeats), and it relates two diseases if any of their disease-proteins share at least one of these signatures.

We noticed that certain MeSH terms correspond to Pfam signatures, and this fact could introduce a bias in the evaluation. With an automated analysis of MeSH terms followed by manual curation, we found 113 descriptors that correspond exactly to a Pfam signature (shown in **Table 2**). We then excluded from our evaluation any disease pair in which a protein's Pfam signature matched any of the ones found in MeSH.

This results in 33,660 pairs relating 2,647 OMIM diseases.

Table 2. Excluded Pfam signatures.

D001081	APYRASE	D004815	EGF
D013049	SPECTRIN	D005914	GLOBIN
D005294	FERROCHELATASE	D014168	TRANSFERRIN
D002364	CASEIN	D005801	HOMEOBOX
D050600	SNARE	D015847	IL4
D051348	TROPOMODULIN	D014357	TRYPSIN
D043169	ENDOSTATIN	D017370	IL11

D002155	CALSEQUESTRIN	D009320	ANP
D001119	ARGINASE	D052243	RESISTIN
D035561	TFIIA	D013884	RHODANESE
D018664	IL12	D005420	FLAVOPROTEIN
D000519	MELIBIASE	D019409	IL15
D016596	VINCULIN	D053673	GLYPICAN
D046988	PROTEASOME	D014216	TAN
D064451	HEPCIDIN	D013004	SOMATOSTATIN
D051152	CLUSTERIN	D008049	LIPASE
D006466	HEMOPEXIN	D014559	UROCANASE
D053523	AMELOGENIN	D019922	NEUROMODULIN
D016547	KINESIN	D035581	TFIIB
D050683	SYNAPTOBREVIN	D025481	6PF2K
D001839	BOMBESIN	D013879	THIOREDOXIN
D003094	COLLAGEN	D013947	THYMOSIN
D052116	ENDOMUCIN	D014598	UTEROGLOBIN
D005293	FERRITIN	D054477	GLUTAREDOXIN
D018793	IL13	D014442	TYROSINASE
D037282	CALRETICULIN	D064248	GEMININ
D018969	IGFBP	D051843	SYNUCLEIN
D025801	UBIQUITIN	D020932	NGF
D016173	CSF-1	D014404	TUBULIN
D056489	NUCLEOPLASMIN	D008084	LIPOXYGENASE
D020738	LEPTIN	D018260	GELSOLIN
D051100	GDNF	D055572	CERAMIDASE
D002966	CLATHRIN	D050765	SYNTAXIN
D015087	CNPASE	D016232	ENDOTHELIN
D051190	PLECTIN	D016633	APOA-II
D050777	STATHMIN	D014198	TREHALASE

D007372	INTERFERON	D005755	GASTRIN
D051304	PROFILIN	D006657	HISTONE
D015848	IL5	D001053	APOLIPOPROTEIN
D011228	TRANSTHYRETIN	D020934	CNTF
D053495	OSTEOPONTIN	D064249	SECURIN
D014335	TROPOMYOSIN	D002148	CALDESMON
D013285	STRABISMUS	D054834	LIPOCALIN
D007376	IL2	D014153	TRANSALDOLASE
D018031	CONNEXIN43	D016753	IL10
D002374	CATALASE	D015851	IL7
D054804	CATHELICIDINS	D004970	FOLLICULIN
D003459	CRYSTALLIN	D014336	TROPONIN
D000199	ACTIN	D064448	FILAMIN
D005972	GLUTAMINASE	D051419	PAXILLIN
D016209	IL8	D006422	HEMERYTHRIN
D053667	SYNDECAN	D064067	RHOGEF
D015850	IL6	D020663	RASGEF
D019004	GALANIN	D007987	GNRH
D053763	PRESENILIN	D001618	BETA-LACTAMASE
D007328	INSULIN	D000516	ALPHA-AMYLASE
		D000154	ACONITASE

PPI dataset

The second relationship presented by Van Driel *et. al.* determines molecular relatedness based on protein-protein interactions between disease proteins. Two diseases are related if their disease proteins interact according to the Human Protein Reference Database (HPRD). This criterion resulted in 15,515 disease pairs relating 2,512 OMIM diseases.

Sequence similarity dataset

The last relationship proposed by van Driel et al. is based on sequence similarity, and it relates two diseases whenever their disease proteins are similar in sequence. Sequence similarity is determined with a Smith-Waterman local alignment of the sequences with a threshold e-value smaller or equal to 10^-6. This criterion results in 37,486 diseases pairs relating 2,817 OMIM diseases.

Finally, in order to avoid any danger of circular reasoning, we have excluded from the evaluation all pairs of diseases which share at least a disease-protein from all three relationships/datasets.

9. Using the MeSH ontological structure improves the accuracy of disease similarity calculations

An important question was whether the use of the ontological structure was necessary for accurate diseases similarity calculations. In order to shed light on this issue, we analysed the performance and some important characteristics of the simple similarity measures detailed in Section 5 of this Supplementary material.

First, the scores produced by the simple similarity measures do not depend on the specificity of the annotations. That is, the quality of the annotations of the individual diseases is not considered and this results in a measure with a reduced capability to discriminate between well-annotated diseases and those annotated with very general but overlapping terms. As an example, 5 overlapping but very general terms are as good as 5 very specific overlapping terms. These measures are "coarse", in the sense that they are unable to determine similarity between any two pairs of *slightly* similar diseases. When the evidence is abundant these measures produce accurate similarity scores. The ontological similarities measures are able to distinguish more nuanced similarity values, particularly Resnik [4] (used by our method) , simUI and simGIC [7] as they are not affected by the problems of Lin's and Jiang and Conrath's measures mentioned in Section 8 of this supplementary material.

Second, while many diseases share annotations, most do not. This lack of annotations is not a definite measure of dis-similarity between diseases as it might simply reflect the lack of valid annotations for this particular pair of diseases conflating the meaning of **zero**

similarity. That is, a zero would at the same time a lack of annotations for diseases and dis-similarity. In contrast, the ontological similarity measures (Resnik, Lin, Jiang and Conrath, simUI and simGIC) are only able to produce a similarity score of **zero** when the root of the ontology is chosen as the Lowest Common ancestor or, equivalently, when two diseases **have no common terms along the path to the root**. Conceptually, such a situation would arise only when two diseases are annotated with terms that are distant from one another in the ontology.

Figure 3a presents a comparison of Resnik's semantic similarity measure used by our method to quantify disease similarities on MeSH, and the simple similarity measures.

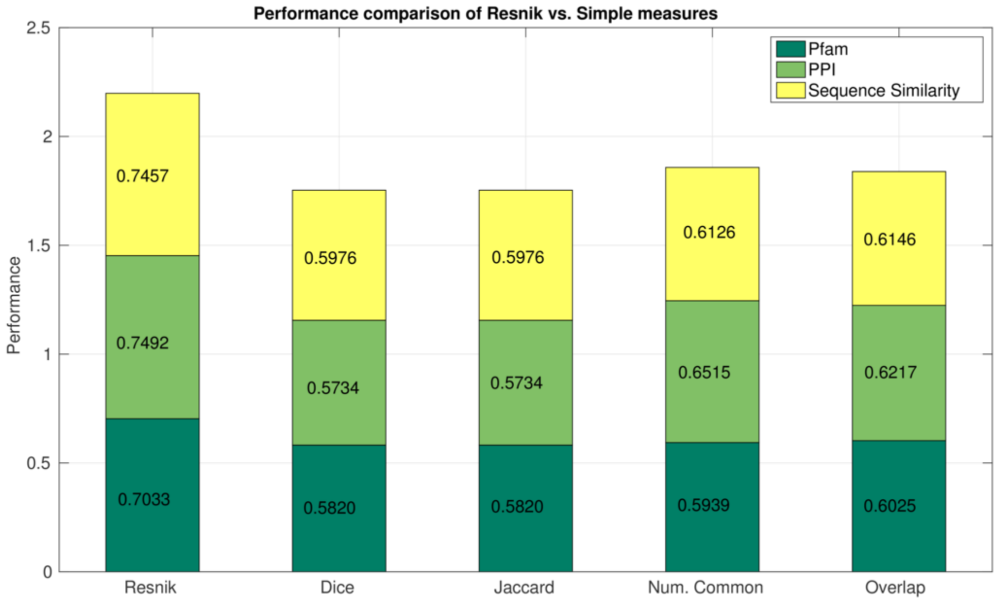

Figure 3a Performance comparison of Our Method, which uses Resnik's measure, and the simpler similarity measures. This figure shows the advantages of using MeSH's ontology structure to calculate smilarity between diseases.

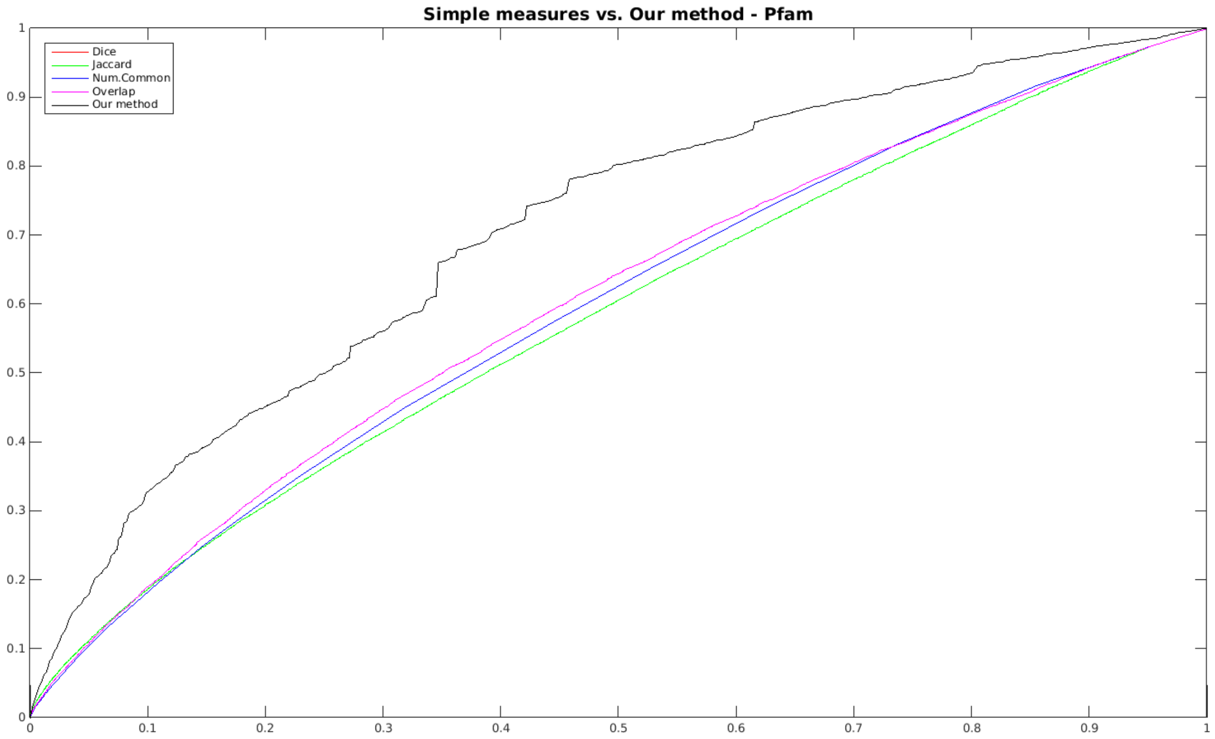

Figure3b ROC plot comparing the performance of Our Method (which uses Resnik's similarity measure) and the simple similarity measures on the Pfam dataset.

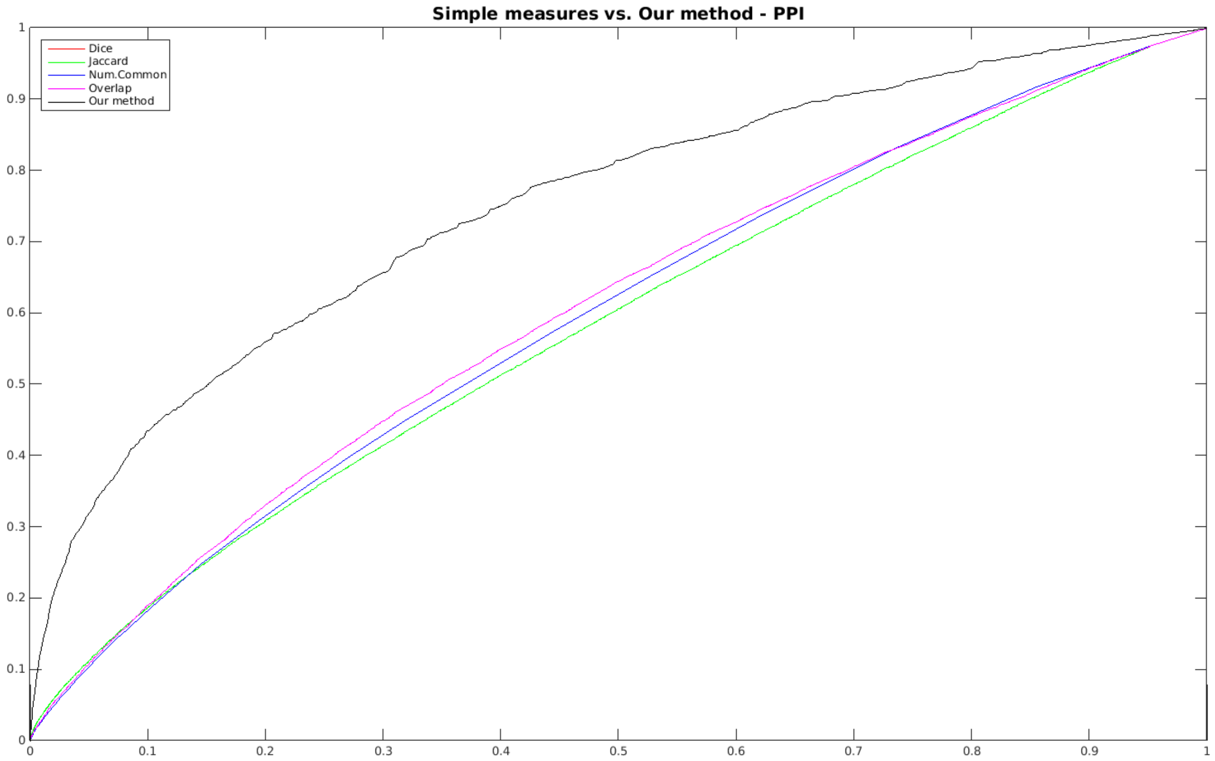

Figure3c ROC plot comparing the performance of Our Method (which uses Resnik's similarity measure) and the simple similarity measures on the Pfam dataset

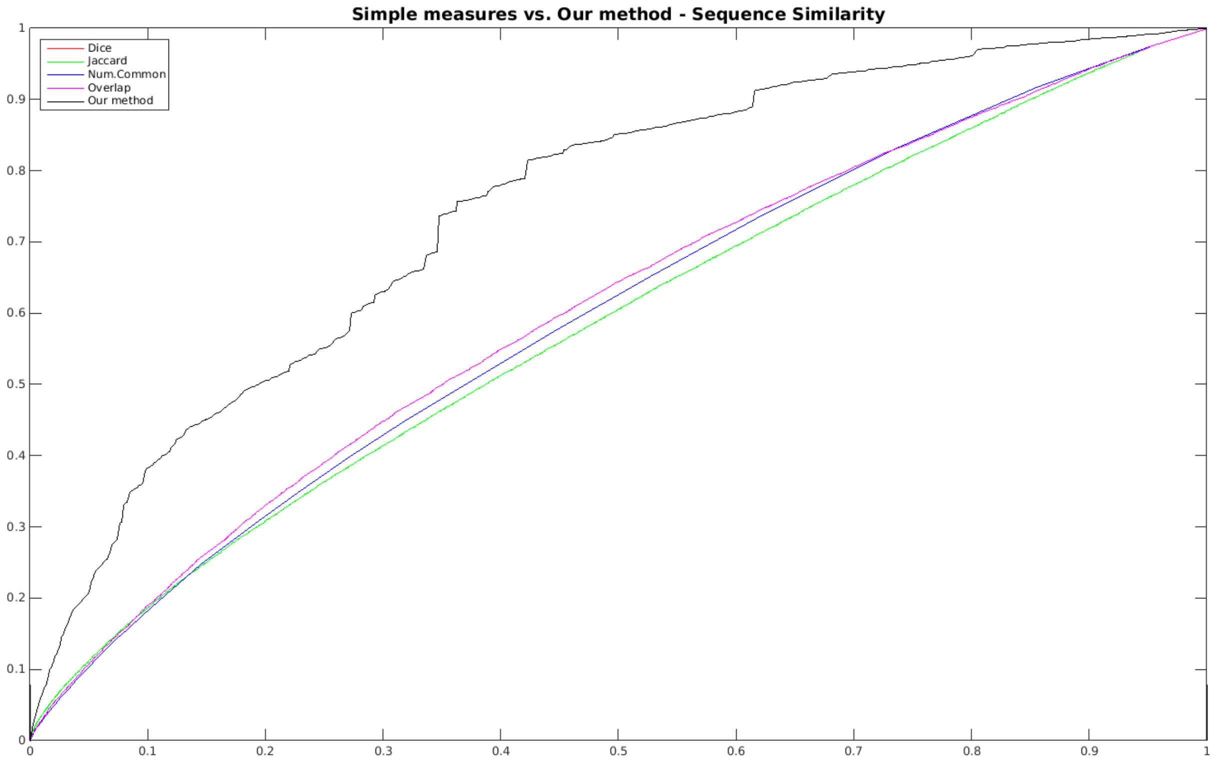

Figure3c ROC plot comparing the performance of Our Method (which uses Resnik's similarity measure) and the simple similarity measures on the Pfam dataset.

These observations coupled to the good performance of Resnik's similarity measure allow us to confidently choose it over the simple similarity measure to calculate disease similarities on MeSH.

In light of the choice of Resnik's measure over the simple similarity measures in combination with its good performance in comparison to the other ontological similarity measures (See section 8 of this Supplementary Material) we have chosen Resnik's measure to calculate disease similarities in MeSH.

10. Correct use of the MeSH ontological structure is essential for accurate disease similarity calculations

While we show that the use of the ontological structure improves accuracy significantly, it is important to note that to fully take advantage of the quality of the annotations, the ontology must be used appropriately.

Several similarity measures are available (Section 4 of this Supplementary Material), however, not all perform equally well. **Figure 4** shows a comparison of the semantic

similarity measures evaluated, were we readily verify that Resnik's similarity measure outperforms all others.

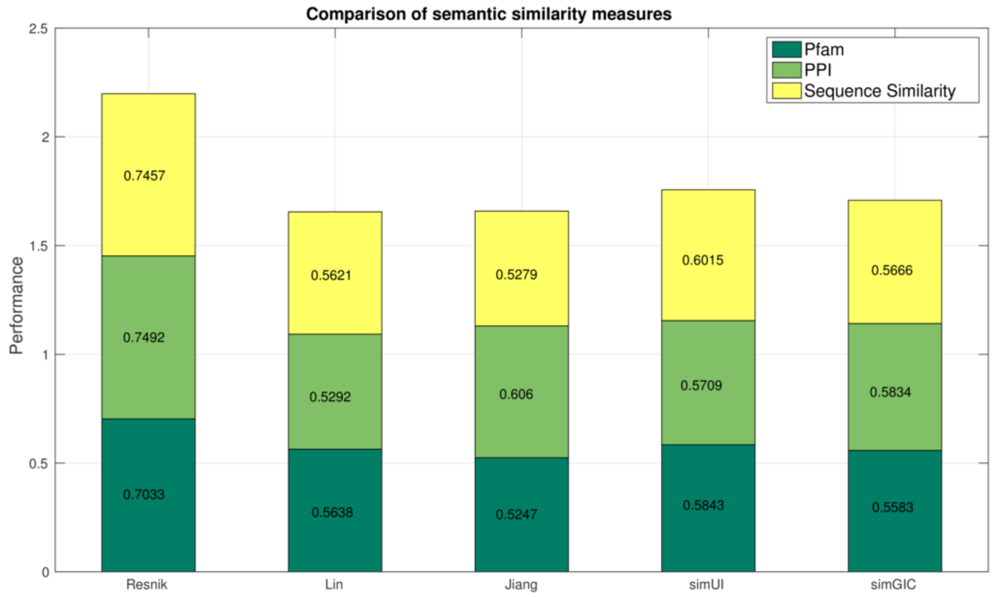

Figure 4 Comparison of the performance for different semantic similarity measures. This figure shows how Resnik's measure (used by our method) makes better use of MeSH's ontology structure.

However, considering that Lin's [5] and Jiang and Conrath's [6] semantic similarity measures are similar to Resnik's, they require further analysis (See Section 4 for a brief introduction on the similarity measures). If two diseases have at least on term in common their similarity will be maximal (*i.e.* 1) irrespective of the specificity of this common term and the number and specificity of the non-overlapping annotations. To illustrate this scenario, consider two diseases annotated as follows: $D_a = \{\mathrm{t1}, \mathrm{t2}, \mathrm{t4}, \mathrm{t6}\}$ and $D_b = \{t1\}$. The similarity of these diseases is 1 given by the similarity of t1 with itself, computed using either Jiang or Lin. A similarity measure that assigns the maximum possible similarity based on this "common annotation" criteria will result in a large proportion of high-similarity pairs whenever high overlap in the annotations exists.

To verify the impact of the overlap, and considering the success of these similarity measures in the context of the Gene Ontology [3, 14] , we analysed the annotations of the model organisms: *A. thaliana, H. sapiens, M. musculus, S. cerevisiae* and *C. elegans*. We extracted the *experimental* annotations for these organisms and counted the overlap which was

compared with the overlap of the annotations produced by our proposed method. The comparison is shown in **Figure 5**

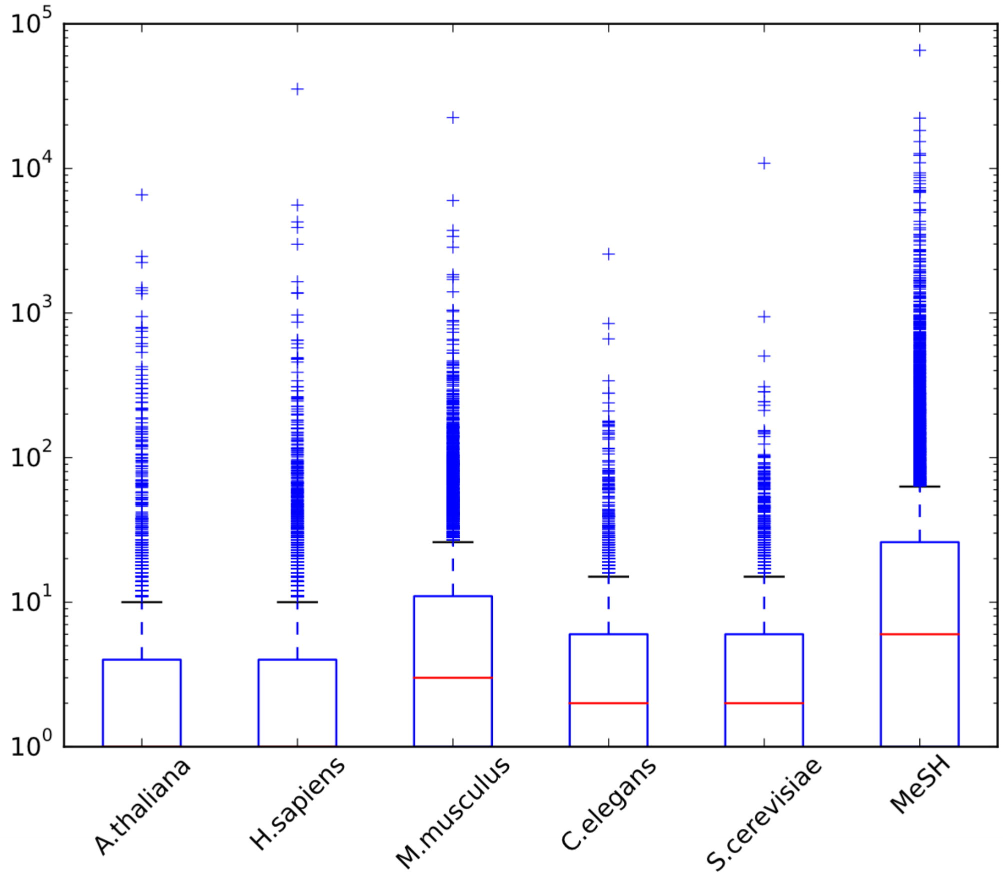

Figure 5 Comparison of the overlap of MeSH annotations in OMIM and GO annotations in the model organisms. The red line in each box corresponds to the median number of times a term is used. The top and bottom parts of each box represent the upper and lower quartile of the distribution. The GO annotations overlap significantly more (t test pvalue < 10-350) than the MeSH annotations. The larger overlap of terms in MeSH makes Lin and Jiang unsuitable to compute OMIM disease similarities in MeSH.

Based on these considerations, we have selected Resnik's semantic similarity measure to calculate disease similarity measures on MeSH.

11. The choice of MeSH term sets: All terms vs. Major topics

MeSH categorises the terms associated to a publication into Major Topic and non-Major Topic. A "MajorTopics" term designates a term extracted from the title or statement of purpose of the publication, and refers to the central focus point. The remaining MeSH terms are either qualifiers for the MajorTopics or refer to topics substantially discussed in the publication.

The coverage of our method was lower when considering only the reduced set of annotations, with 7,094 (90.8% of OMIM) of the diseases having associated *MajorTopics* vs 7,575 (96.8% of OMIM) when considering *AllMeSH*. At the same time, the performance was similar. Therefore, for our methods we chose to use the full set of MeSH terms.

12. Performance of the measure in the individual ontologies

Figures 8-22 show the performance of our method for each individual ontology on the three relationships PPI, Pfam and Sequence Similarity described earlier.

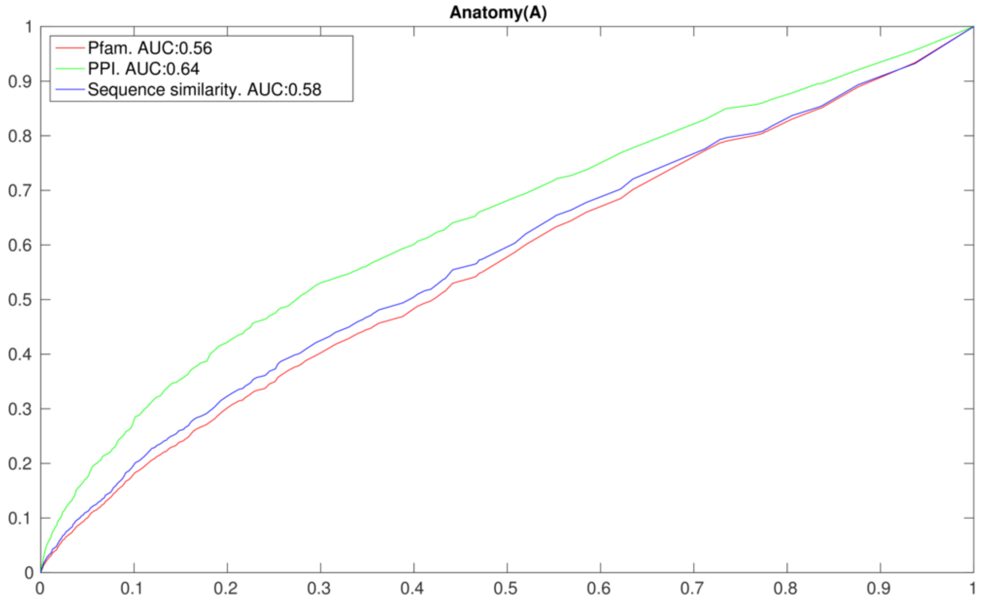

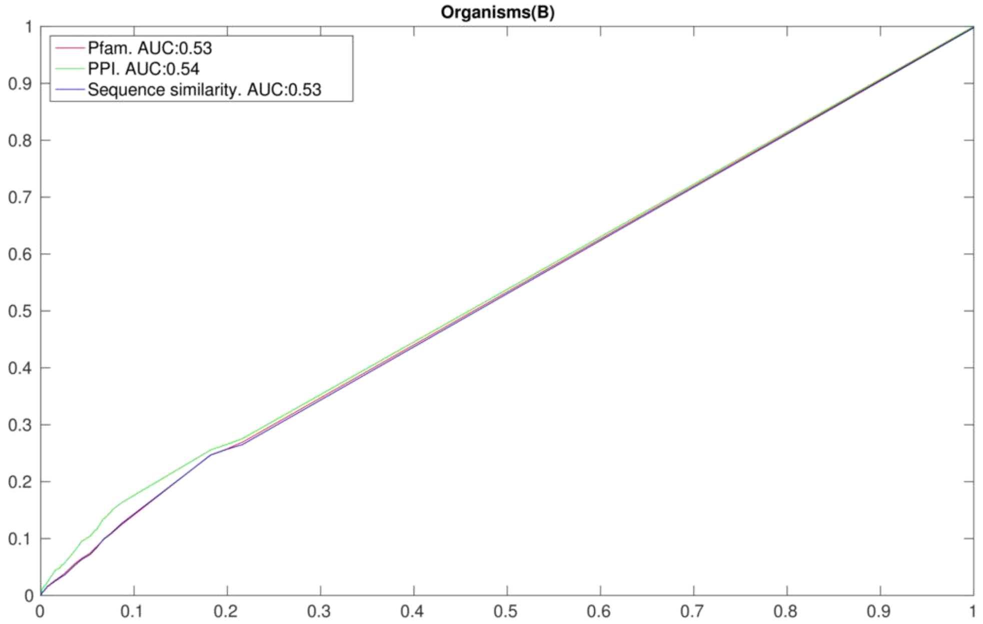

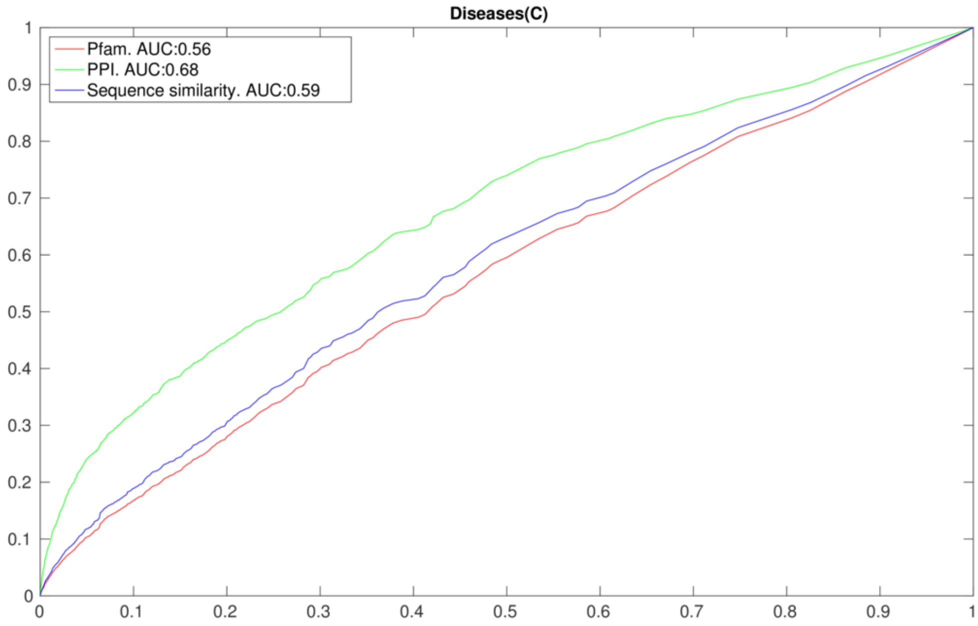
Diseases(C)
Pfam. AUC:0.56
PPI. AUC:0.68
Sequence similarity. AUC:0.59
0
0.1
0.2
0.3
0.4
0.5
0.6
0.7
0.8
0.9
1

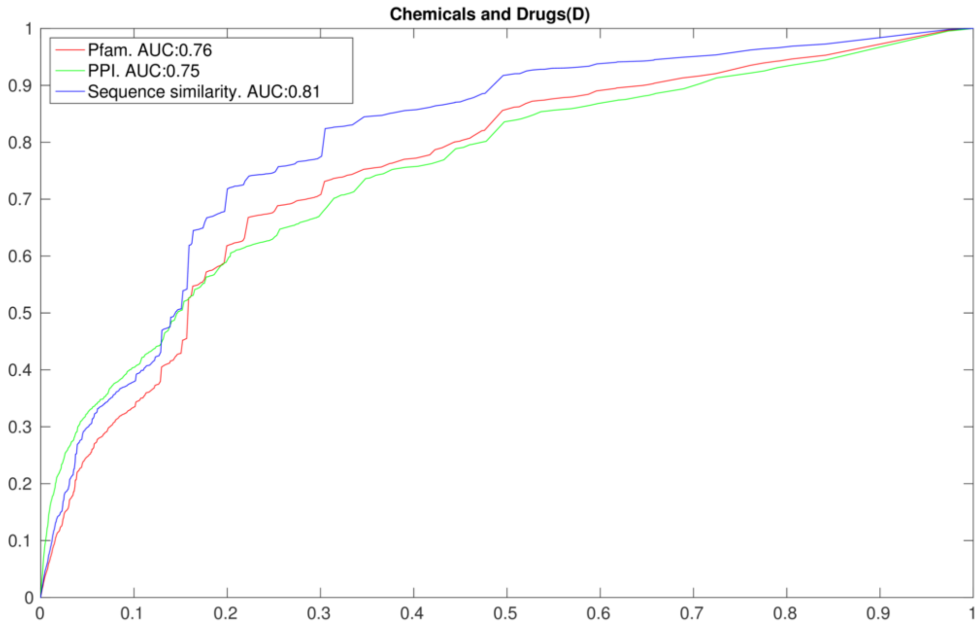
Chemicals and Drugs(D)
Pfam. AUC:0.76
PPI. AUC:0.75
Sequence similarity. AUC:0.81
0
0.1
0.2
0.3
0.4
0.5
0.6
0.7
0.8
0.9
1

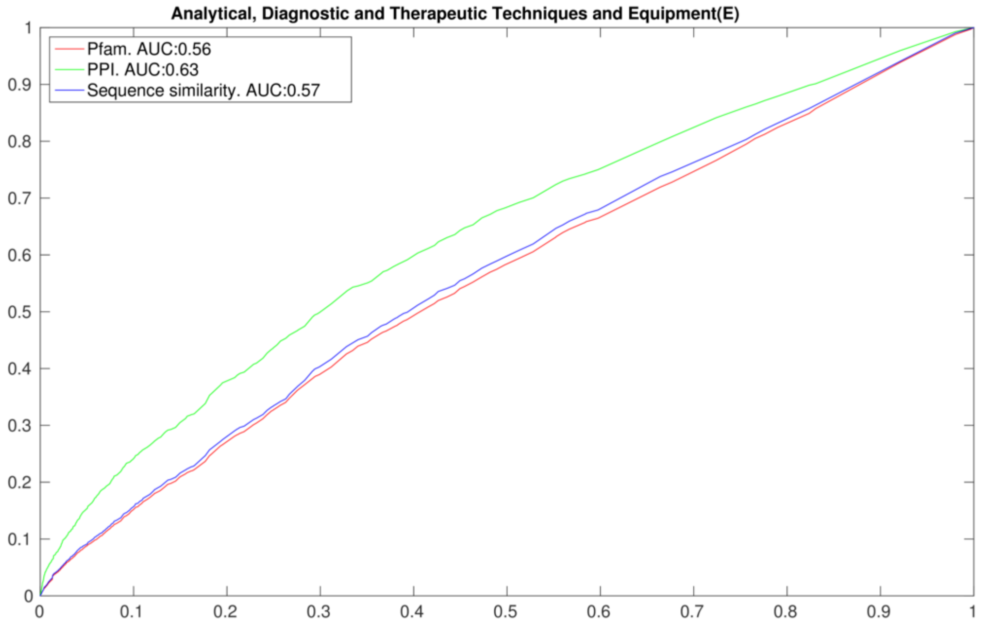
Analytical, Diagnostic and Therapeutic Techniques and Equipment(E)
Pfam. AUC:0.56
PPI. AUC:0.63
Sequence similarity. AUC:0.57

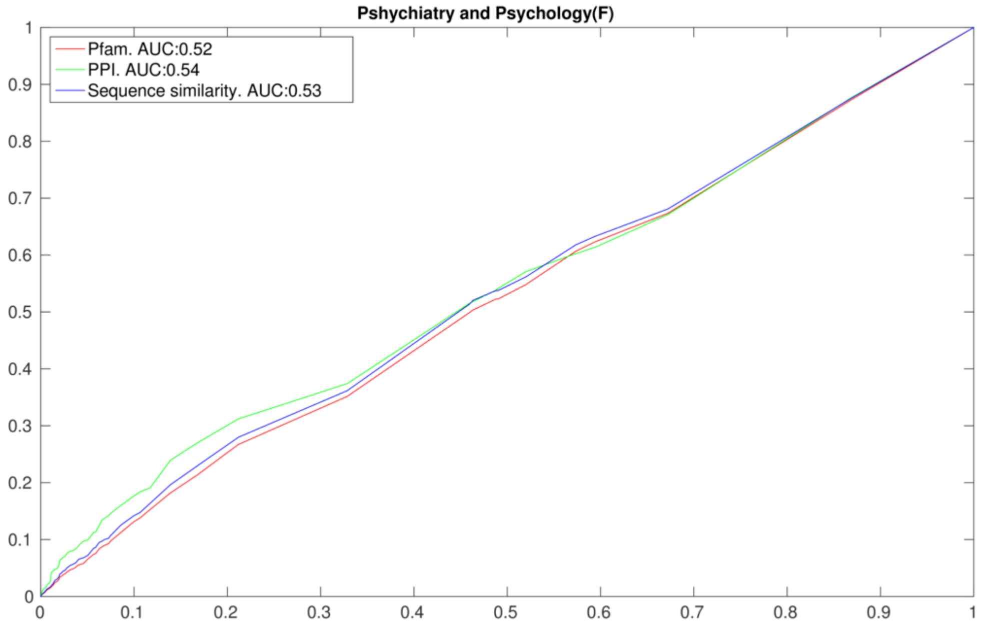
Pshychiatry and Psychology(F)
Pfam. AUC:0.52
PPI. AUC:0.54
Sequence similarity. AUC:0.53

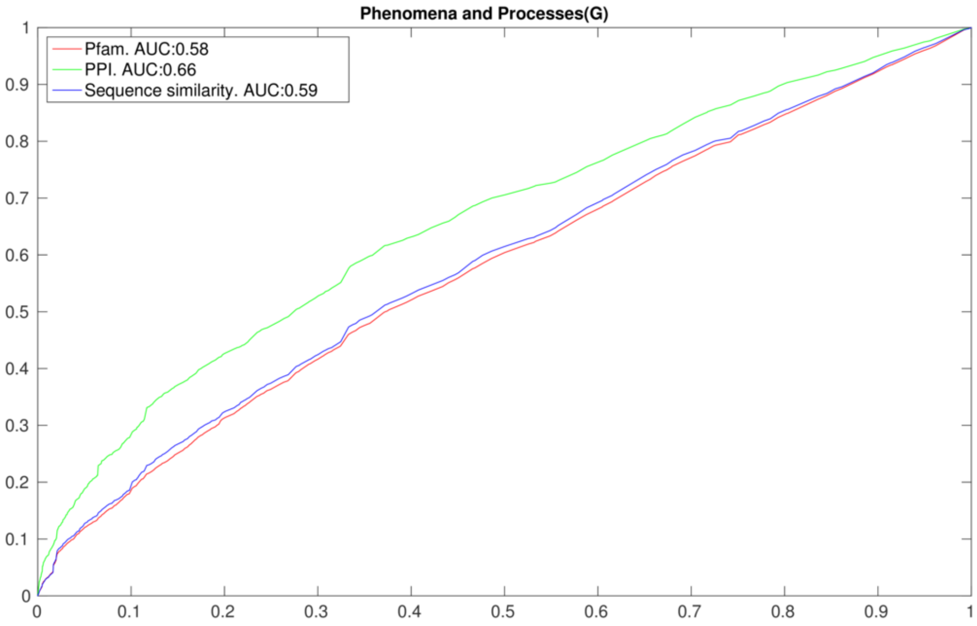
Phenomena and Processes(G)
Pfam. AUC:0.58
PPI. AUC:0.66
Sequence similarity. AUC:0.59
0
0.1
0.2
0.3
0.4
0.5
0.6
0.7
0.8
0.9
1

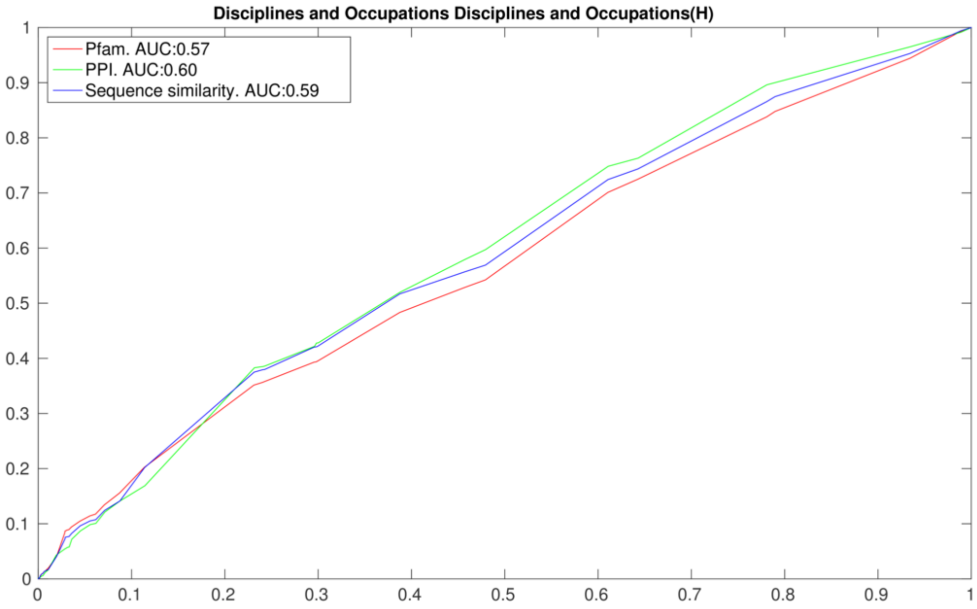
Disciplines and Occupations Disciplines and Occupations(H)
Pfam. AUC:0.57
PPI. AUC:0.60
Sequence similarity. AUC:0.59
0
0.1
0.2
0.3
0.4
0.5
0.6
0.7
0.8
0.9
1

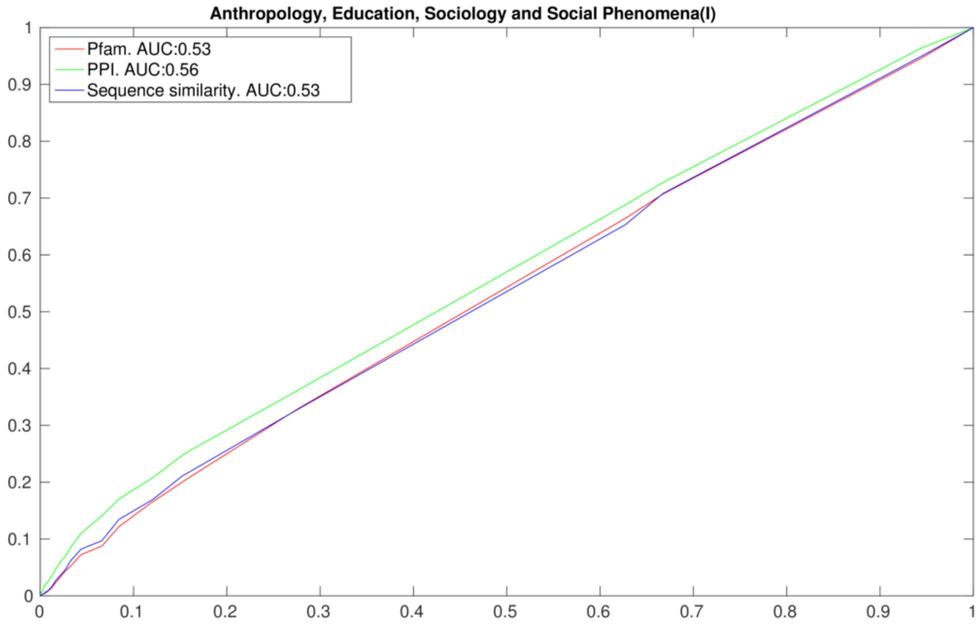
Anthropology, Education, Sociology and Social Phenomena(I)
Pfam. AUC:0.53
PPI. AUC:0.56
Sequence similarity. AUC:0.53
0
0.1
0.2
0.3
0.4
0.5
0.6
0.7
0.8
0.9
1

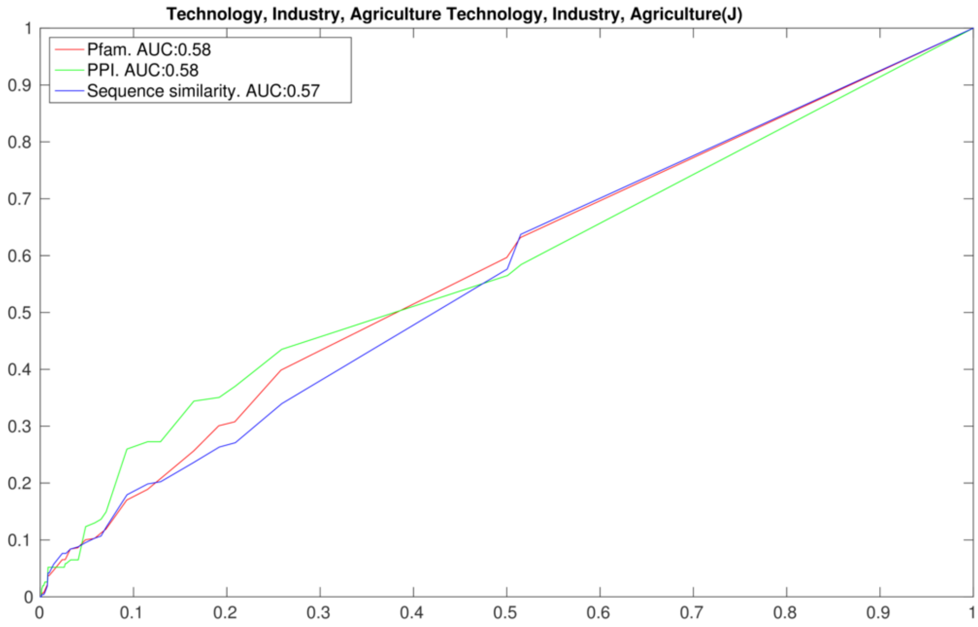
Technology, Industry, Agriculture Technology, Industry, Agriculture(J)
Pfam. AUC:0.58
PPI. AUC:0.58
Sequence similarity. AUC:0.57
0
0.1
0.2
0.3
0.4
0.5
0.6
0.7
0.8
0.9
1

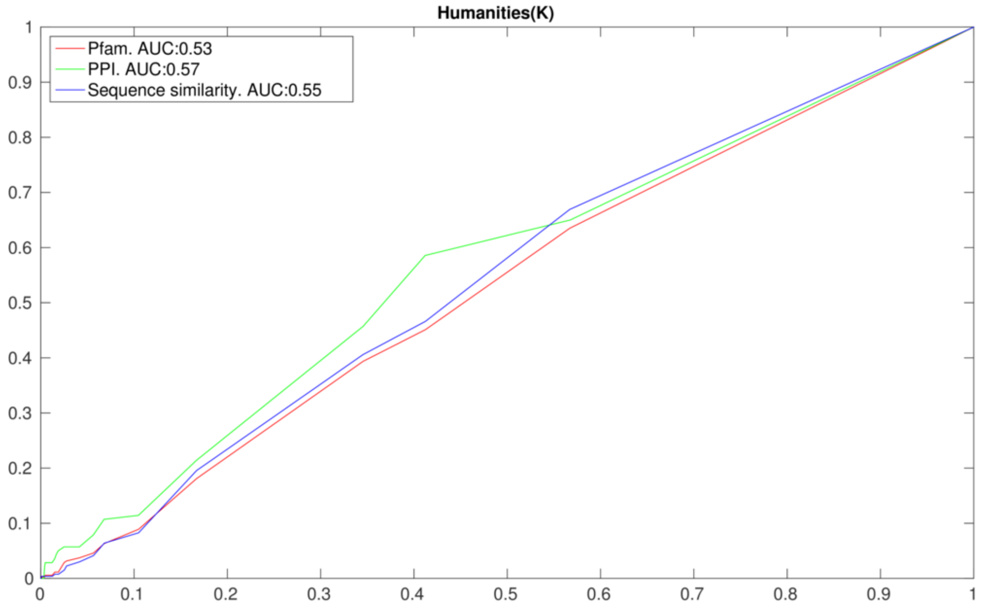
Humanities(K)
Pfam. AUC:0.53
PPI. AUC:0.57
Sequence similarity. AUC:0.55
1
0.9
0.8
0.7
0.6
0.5
0.4
0.3
0.2
0.1
0
0
0.1
0.2
0.3
0.4
0.5
0.6
0.7
0.8
0.9
1

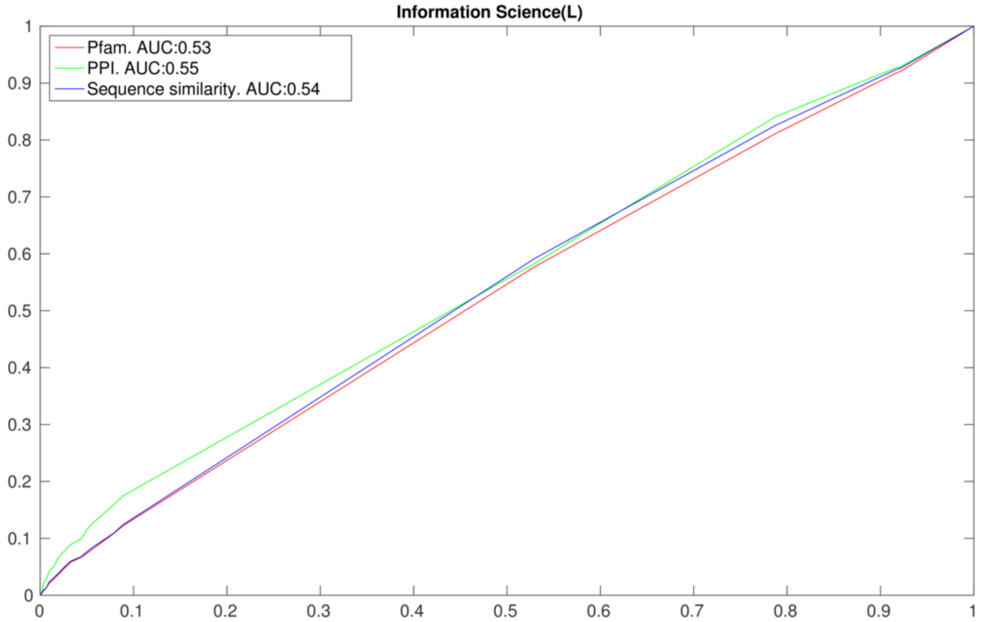
Information Science(L)
Pfam. AUC:0.53
PPI. AUC:0.55
Sequence similarity. AUC:0.54
1
0.9
0.8
0.7
0.6
0.5
0.4
0.3
0.2
0.1
0
0
0.1
0.2
0.3
0.4
0.5
0.6
0.7
0.8
0.9
1

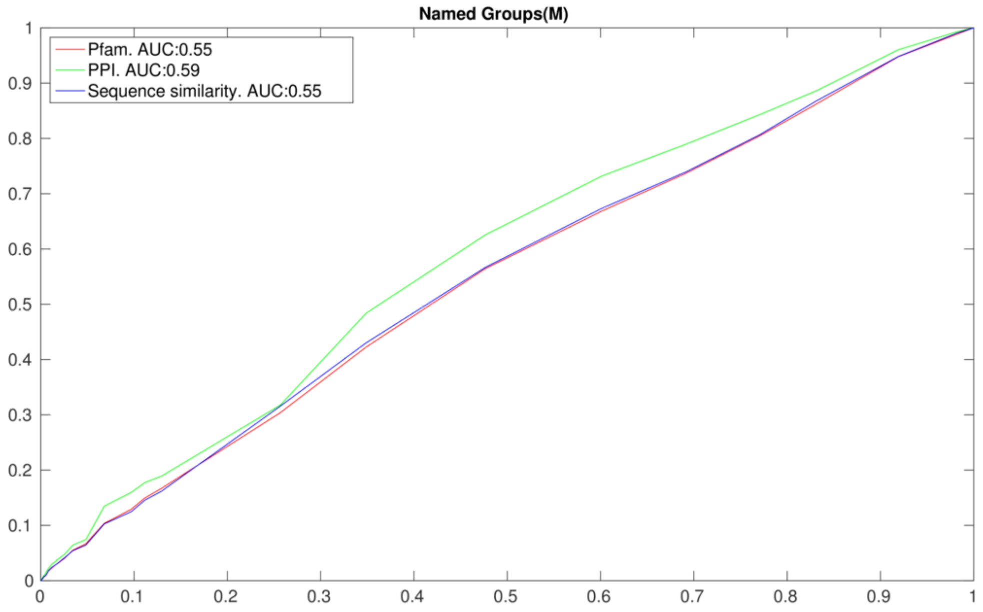
Named Groups(M)
Pfam. AUC:0.55
PPI. AUC:0.59
Sequence similarity. AUC:0.55
0
0.1
0.2
0.3
0.4
0.5
0.6
0.7
0.8
0.9
1

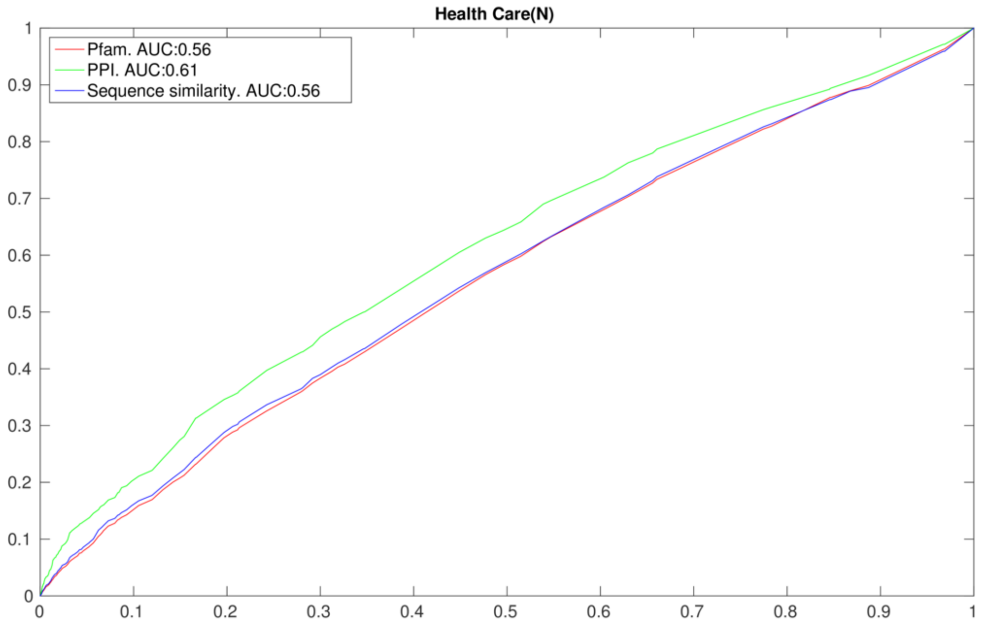
Health Care(N)
Pfam. AUC:0.56
PPI. AUC:0.61
Sequence similarity. AUC:0.56
0
0.1
0.2
0.3
0.4
0.5
0.6
0.7
0.8
0.9
1

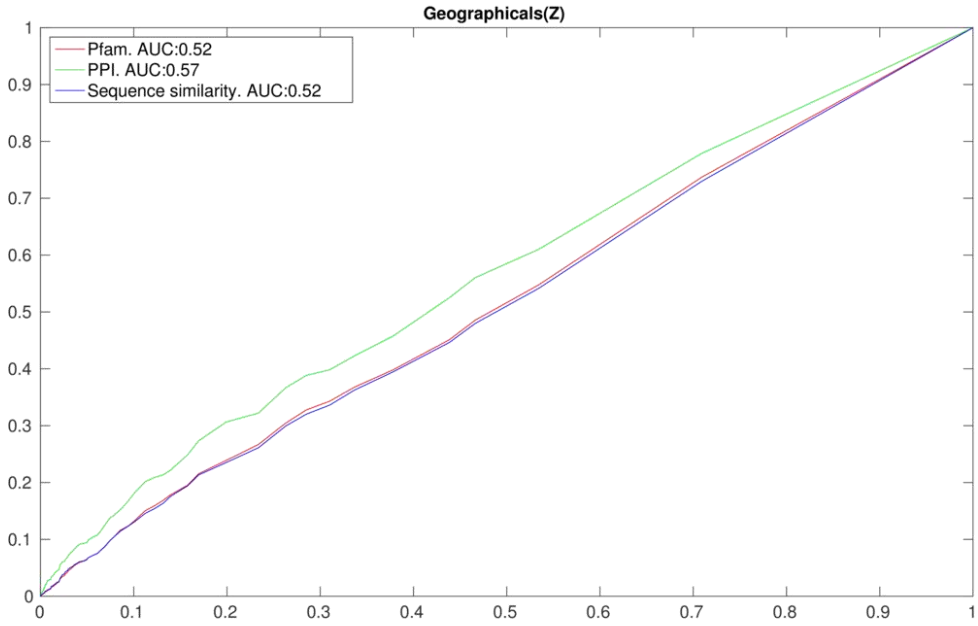

13. Combining the MeSH ontologies. Performance plots.

It is important to note that there are 16 Ontologies in MeSH, thus the similarity method described so far will result in up to 16 similarity scores for each pair of diseases. Since our aim is to produce a single similarity score for each pair of diseases, we need to combine the ontologies.

Our analysis of MeSH revealed a large overlap between the various ontologies. Nevertheless, note that the most relevant factor in the combination is the existence of paths between the different ontologies, not only on the overlap. As shown in **Figure 23** (reproduced from the main paper), the shared terms connect most ontologies.

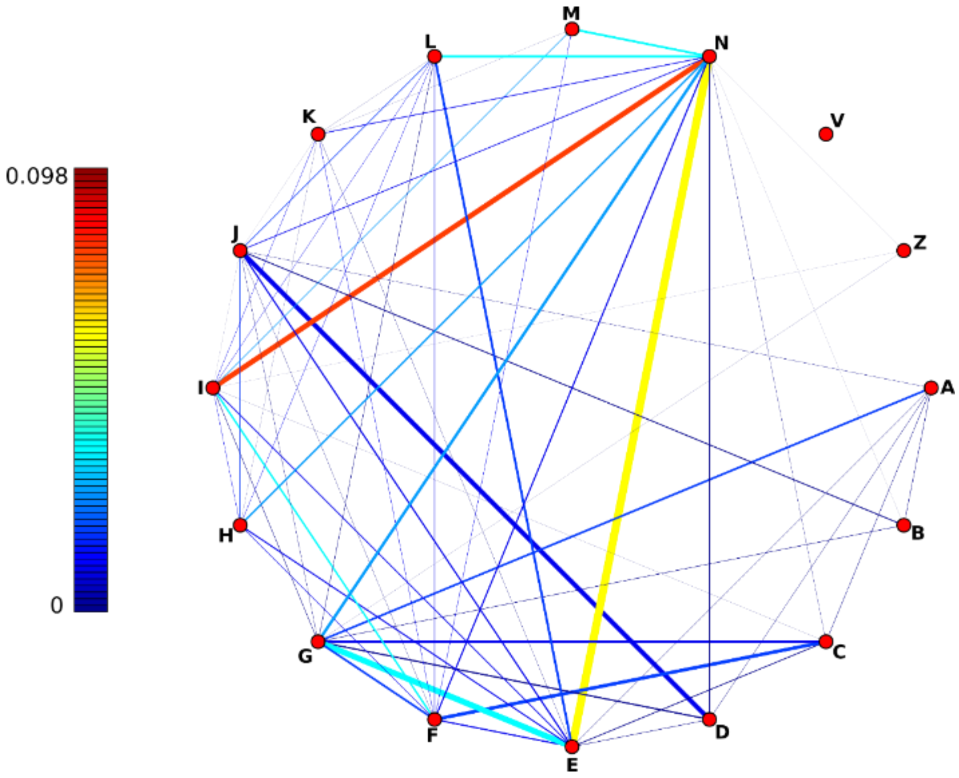

Figure 23. Overlap of the MeSH ontologies. Nodes represent MeSH ontologies and links are related to the amount of overlap between them. Link colours correspond to the Jaccard coefficient between the set of terms in each pair of ontologies. Link thicknesses correspond to the number of shared terms between ontologies and only strictly positive links are shown. MeSH Ontologies abbreviations: [A] Anatomy, [B] Organisms, [C]Diseases, [D] Chemicals and drugs, [E] Analytical, Diagnostic and Therapeutic Techniques and Equipment, [F] Psychiatry and Psychology, [G] Phenomena and Processes, [H] Disciplines and Occupations, [I] Anthropology, Education, Sociology and Social Phenomena, [J] Technology, Industry, Agriculture, [K] Humanities, [L], Information Science, [M] Named Groups, [N] Health Care, [V] Publication Characteristics, [Z] Geographical.

This interconnectedness allows us to combine the ontologies in a simple way. By adding a fictitious node at the top level connecting to all first level nodes from of the MeSH ontologies to be combined, we are able to maintain the ontological structure and obtain a single comprehensive ontology. When used, this combined ontology results in a single score for each pair of disease. **Figure 24** shows the paths and the fictitious root node in a toy example. Terms labelled t1 and t2 illustrate two terms used to annotate diseases.

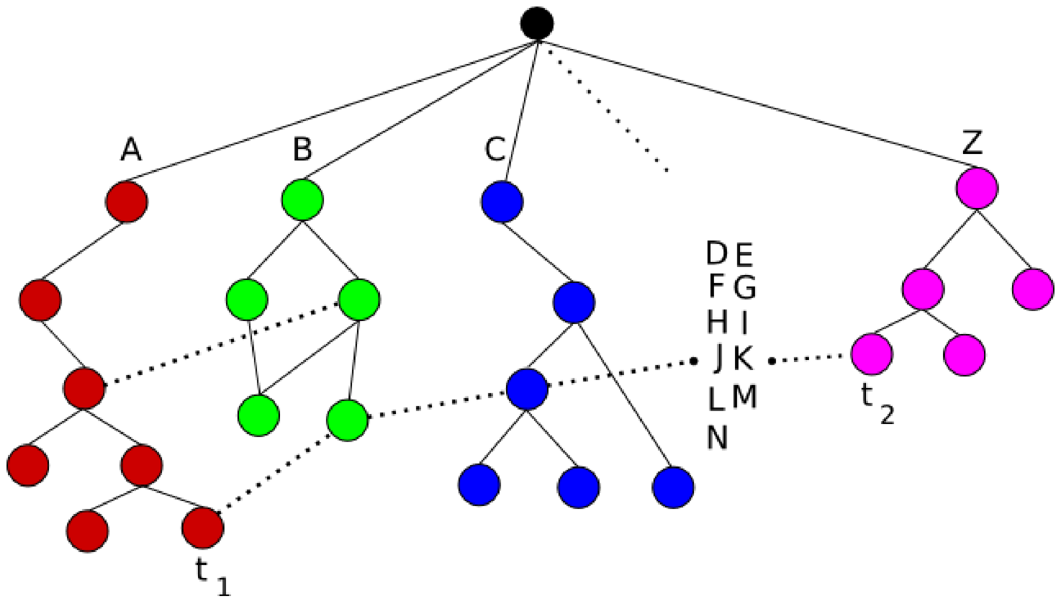

Figure 24 Paths between overlapping ontologies. The overlap between the ontologies allows the up propagation of information across ontologies.

At this point, having identified the need and procedure to combine the ontologies, we chose to combine the A, C, D, E and G ontologies. **Table 3** shows the number of times terms in each ontology that are used to annotate an OMIM disease. Based on this table, we can safely discard the V (Publication characteristics) ontology, which is not used to annotate any diseases.

Table 3. Usage of terms in each ontology

Ontology	Annotated diseases	AUC Pfam	AUC PPI	AUC Sequence
A	83,081	0.56	0.64	0.58
B	89,366	0.53	0.54	0.53
C	160,532	0.56	0.68	0.59
D	142,106	0.76	0.75	0.81
E	108,855	0.56	0.63	0.57
F	15,978	0.52	0.54	0.53
G	197,657	0.58	0.66	0.59
H	4,945	0.57	0.60	0.59
I	4,024	0.53	0.56	0.53

J	638	0.58	0.58	0.57
K	708	0.53	0.57	0.55
L	24,910	0.53	0.55	0.54
M	105,358	0.55	0.59	0.55
N	27,282	0.56	0.51	0.56
V	0	0	0	0
Z	10,869	0.52	0.57	0.52

Analysing the usage and performance of each ontology, we combine the 5 ontologies whose AUC in PPI is above 60% while maintaining a good coverage. We chose the PPI AUC considering that this dataset is the most stringent one. It positively relates far fewer disease than the Pfam (46% smaller) and the Sequence similarity (41% smaller) datasets. We also tried other combinations and we found results to be equivalent as long as we included ontologies with high coverage.

14. Performance comparison of existing methods of disease similarity. ROC plots and Bar charts.

In this section we present the performance for the Pfam, PPI and Sequence similarity datasets for the disease similarity methods presented in the paper. Namely, Our method, van Driel, Park and Robinson. **Figures 25, 26** and **27** presents the ROC plots for the disease similarity for each method

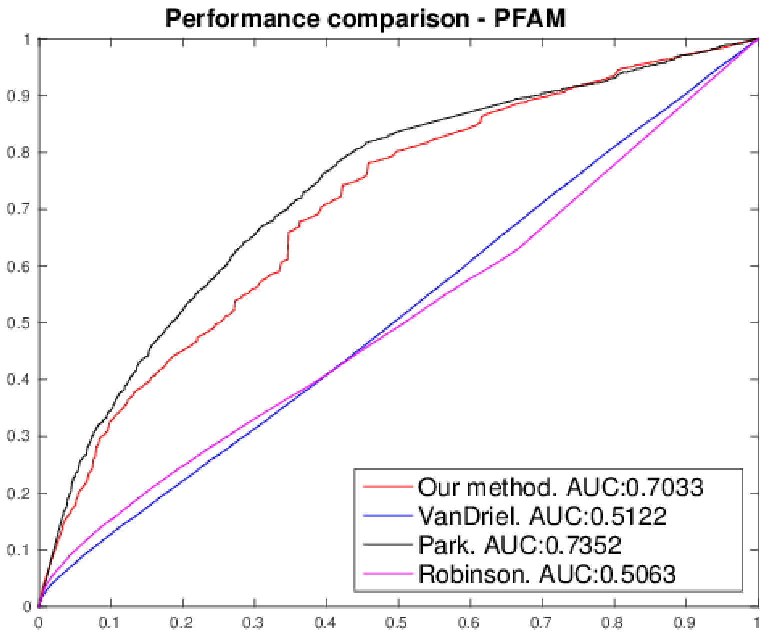

Figure 25 ROC plot PFAM. Performance comparison of the disease similarity measures.

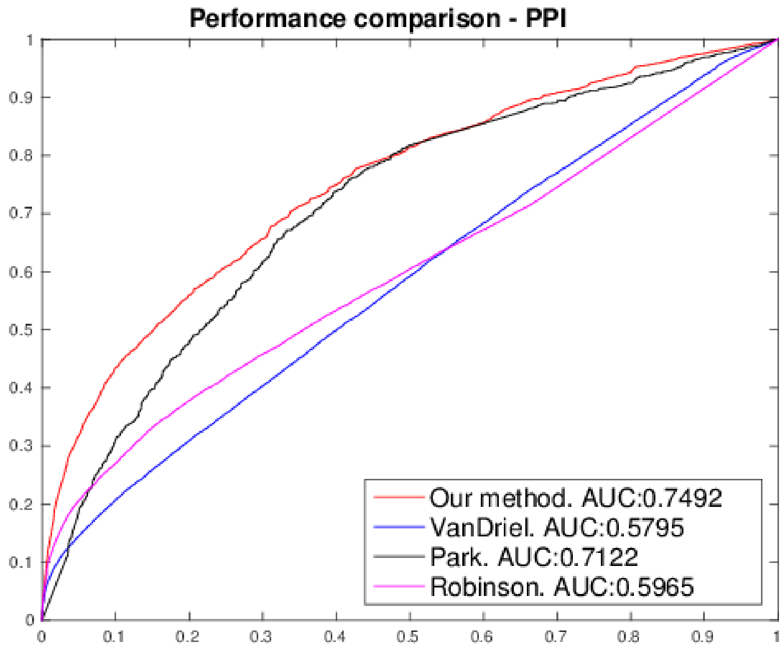

Figure 26 ROC plot PPI. Performance comparison of the disease similarity measures

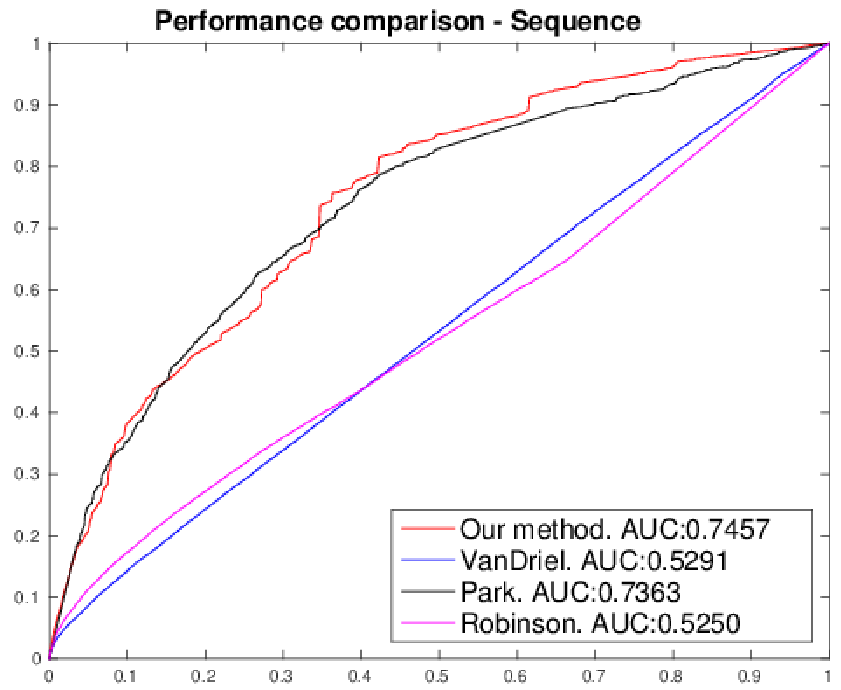

Figure 27 ROC plot Sequence. Performance comparison of the disease similarity measures

Note that the ROC curves do not consider coverage, however, to evaluate the practical importance of any measure, coverage has to be considered. **Figure 28** replicates the figure 1a of the main paper and shows the composite score combining coverage with AUC for each method.

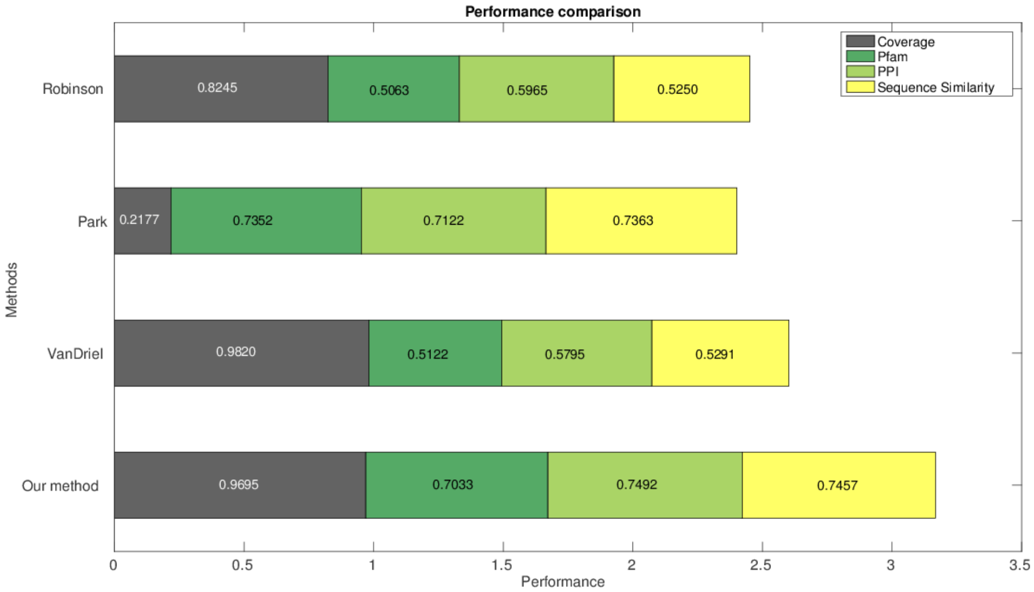

Figure 28 Performance Comparison. This figure is replicated from the main manuscript for completeness. For each method, the grey bar quantifies its OMIM coverage, coloured bars quantify its performance measured by AUCs on the Pfam, PPI and Sequence Similarity datasets. The total length of each bar represents the overall performance of the methods.

15. Ontological similarities in MeSH: A Two-step process

Conceptually, our proposed method can be thought of as a two-step process: an annotation step that results in MeSH terms being assigned to OMIM diseases (i.e. an OMIM-to-MeSH mapping) and a similarity calculation step.

We compare the performance of each individual step in our method with the performance of the equivalent steps in the method proposed by van Driel *et al.* [8]. This was done using the code kindly provided by Prof. Han G. Brunner (Radboud University Nijmegen Medical Centre, Department of Human Genetics).

We have decoupled the steps in both methods as follows:

1. We replace in our pipeline our OMIM-to-MeSH mapping with van Driel's *et al* OMIM-to-MeSH mapping (resulting from the text-mining analysis of the *Clinical Synopsis* and *Text* fields of OMIM). Note that, since our method does not require weights for this initial mapping we remove the weights in van Driel's *et al.* OMIM-to-MESH mapping.

2. Conversely, to verify the similarity calculation step, we altered van Driel's *et.al.* pipeline, replacing their OMIM-to-MeSH mapping with ours. As van Driel's *et.al.* method requires weights and in our annotations duplicate MeSH terms are not considered, a weight of 1 was assigned to each MeSH term.

Figures 29, 30 and **31** show the evaluation results for the Pfam, PPI and Sequence Similarity datasets.

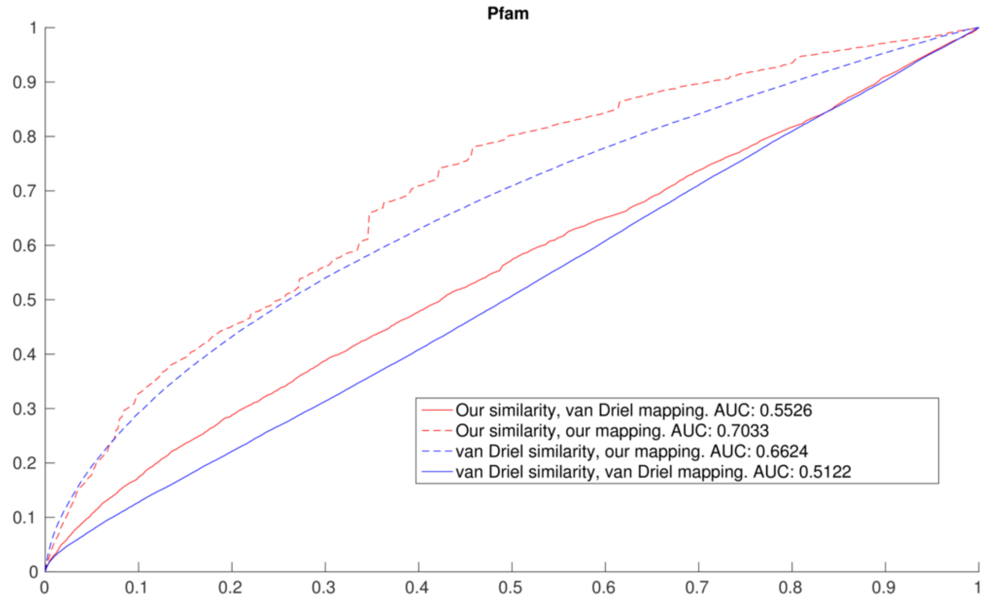

Figure 29 Performance comparison of the OMIM to MeSH mapping (Step 1) and the similarity calculation (Step 2) between our method and van Driel's *et al.* method on the Pfam dataset.

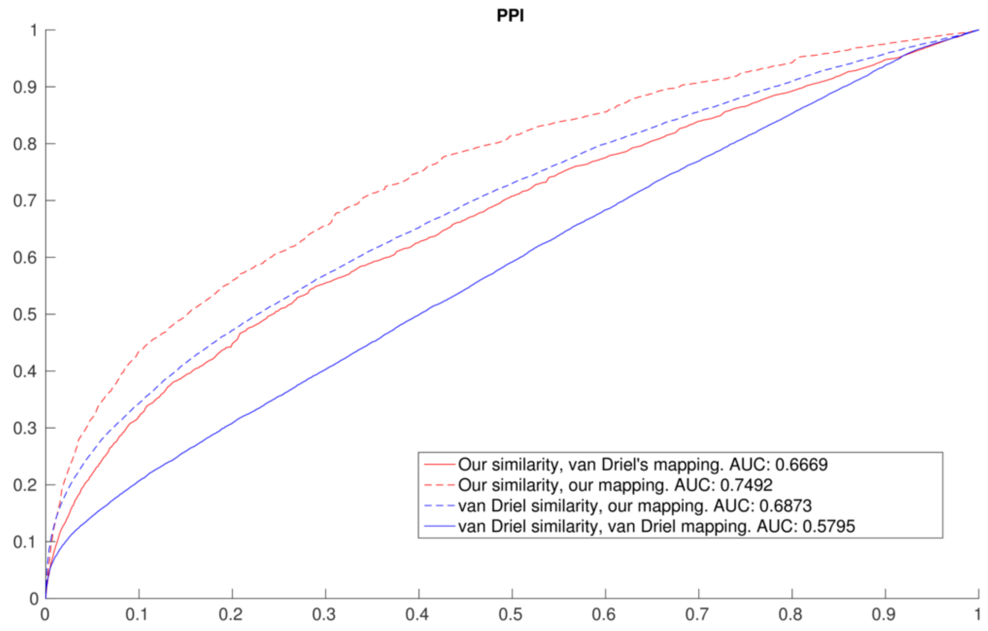

Figure 30 Performance comparison of the OMIM to MeSH mapping (Step 1) and the similarity calculation (Step 2) of our method and van Driel's *et al.* method on the PPI dataset.

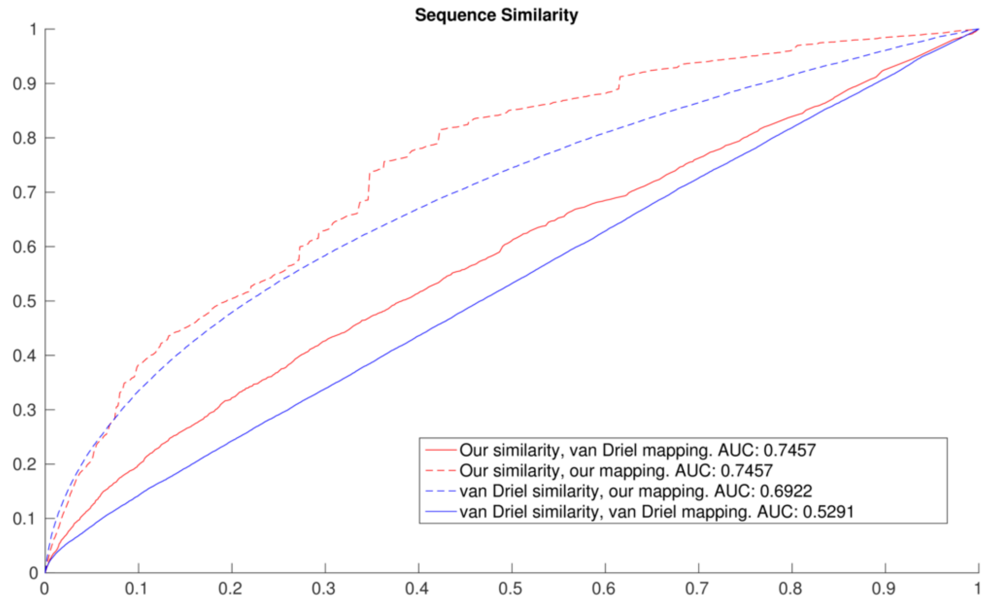

Figure 31 Performance comparison of the OMIM to MeSH mapping (Step 1) and the similarity calculation (Step 2) of our proposed method and van Driel's *et al.* method on the Sequence Similarity dataset.

16. Small variability in scores for highly similar diseases

When analysing highly similar diseases we noted that, in some cases, there is very little variability in the similarity scores. That is, in a relatively large set of disease pairs, very few

different scores are present. As an example, the similarity between Breast Cancer (MIM: 114480) and the 10 diseases most similar to it is shown in **Table 4**. This is due to the fact that the score depends on number of diseases annotating the lowest common ancestor, and it can happen that this number is the same for different pairs of diseases, even if the common ancestor is different. Looking at lowest common for a few examples, we notice that, while the variability of scores is low, the lowest common ancestors (LCA) are different.

Table 4. Breast cancer and the most similar diseases to it

**OMIM Disease**	**Similarity score – Lowest Common Ancestor**
Cervical Cancer (MIM: 603956)	3.4 (D050658 – Core binding factor beta Subunit)
Phosphoglycerate dehydrogenase (MIM: 601815)	3.58 (D050543-Phosphoglycerate Dehydrogenase)
Mammographic density (MIM: 607308)	3.4 (D008327-Mammography)
Episodic Kinesigneic Dyskenisia (MIM: 128200)	3.4 (D002571-Cerumen)
Breast-ovarian cancer (MIM: 604370)	3.58 (D015413-Mastectomy, Simple)
Retinoblastoma (MIM: 180200)	3.58 (D009366-Neoplasm Seeding)
Severe combined immunodeficiency (MIM: 102700)	3.58 (D003641-Deamination)
Estrogen receptor (MIM: 133430)	3.58 (D056921-Nuclear Receptor Coactivator 3)
Epidermolysis bullosa (MIM: 226730)	3.58 (D039503-Integrin alpha6)
Hypertrichosis (MIM: 135400)	3.58 (D000232-Adenofibroma)

In the case of the LCA's chosen for those diseases with score 3.58, they annotate only the diseases in the example and correspond to leafs in the MeSH ontology. The LCA's corresponding to disease pairs with score 3.40 annotate three diseases and, while D000232 and D008327 do not correspond to leaves in the MeSH ontologies their children do not annotate any diseases.

17. Details on the use of *Goh's etal*. Human Disease Network

Mapping OMIM diseases to Syndromes

Through automatic textual comparison of the disease names followed by manual curation, Goh etal combine the individual diseases in OMIM into syndromes. For example, *Anemia, hypochromic microcytic (MIM: 206100)* and *Anemia, hemolytic, Rh-null, regulator type (MIM: 268150)* are combined into a single syndrome. These syndromes are collected in the *curated_morbidmap* file.

In their *curated_morbidmap* file, several syndromes lacked an OMIM number for the phenotype. To find the OMIM number for the incomplete entries, we searched the OMIM *morbidmap* from July 21st 2014. Each incomplete entry was compared to all entries in the *morbidmap* file and when an entry with a Levenshtein string similarity ratio higher than 0.9 was found, that OMIM number was selected. If more than one entry satisfied the similarity criteria, the entry was discarded. The matched entries were verified manually. Of the 1,717 OMIMs extracted from the *curated_morbidmap,* 1,542 OMIMs were directly matched and 175 OMIMs where extracted through string matching with the official *morbidmap*.

Extracting the disease classes

Goh *et.al* classify each syndrome based on the physiological system it affects. For example, Anaemia is a Haematological disorder. A total of 20 disease classes are defined: Bone, Cancer, Cardiovascular, Connective tissue disorder, Dermatological, Developmental, Ear-Nose-Throat, Endocrine, Gastrointestinal, Haematological, Immunological, Metabolic, Multiple, Muscular, Neurological, Nutritional, Ophthalmological, Psychiatric, Renal, Respiratory and Skeletal. All diseases that the authors were unable to correctly classified were labelled "Unclassified".

After extracting the OMIM diseases from the *curated_morbidmap* (see *Mapping OMIM diseases to Syndromes* in this Supplementary material) we extracted their respective classes and obtained a mapping of OMIM diseases to Disease classes. We were able to assign classes to the 1,717 OMIM diseases we extracted from the *curated_morbidmap.*

18. Details on the use of old versions of OMIM

The examples presented in the main paper further illustrate the capabilities of our method. From an older version of OMIM (April 9, 2013) we were able to extract diseases that were

just recently associated to a gene (after July 21, 2014). We then computed the similarity scores using the older version of OMIM and extracted high-similarity pairs to verify the capability of our method at predicting molecular relatedness.

The following table presents the publications available in the old version of OMIM in the form of PubMed identifiers.

187500 – Tetralogy of Fallot	10587520,5065286,20807224,4003436,9132487,11152664,4834778,11714651,19597493,8923932,9188669,4050848,15937089,20631719,20581743,1425789,2260602,21110066,13943847,14517948,19948535,21919901,18055909,18672102
208530 Right Atrial isomerism	6638068,7715640,9152295,874654,3674113,4003441,3425603,8834045,6712272,8873667,7172476,6622295,1021593,6050934,6638069,14929628,2012140,1191445,14128648,4774542,9155619,9443444,7277426
125853 – Diabetes Mellitus, Noninsulin-dependent, NDDIM	17726085,11443197,10973253,16885549,2695375,22286214,9038347,10720052,12874106,18323454,17463246,19657112,15808156,10199785,10331426,11575290,10958757,9745421,15924147,17273962,8528247,12915642,8528248,11032783,21186350,16775236,17603485,12783844,9758619,11130726,18477659,15472205,20016592,7971976,9032096,11158011,19020324,15940393,11030756,1357346,17066296,17906635,19020323,15070960,16142453,11916952,1587533,20085713,20574426,19933169,12750520,14960743,10902787,12045211,9498630,9541507,18952314,12851856,17603484,12118251,15980866,17463248,9312173,9482914,11723072,17179727,17293876,18008060,8897863,9062343,11904371,9892237,18711366,12727978,18231124,11067779,11032784,20360734,17554300,21118154,22456733,16034410,18711367,22456732,11533494,17463249,22456734
269880 – SHORT syndrome	6407320,8574420,8790109,15481036,12514365,18384141,21340693,8279490,8669449,2729352,4050863

607907 – Dermatofibrosarcoma protuberans	9738795,11291071,11435686,12209598,17478383,12202658,12660034,8988177,12661001,15221986
607785 – Juvenile Myelomonocytic leukemia	19420352,18182584,19388938,10086728,19372255,15723289,21562564,19571318,11588050,17332249,20008299,9160658,20543203,9616134,16474405,12717436

19. Analysis of the performance for Complex diseases in OMIM

While there is not definitive way to classify diseases in OMIM as *Complex* or *Mendelian* we attempted three different ways of obtaining this classification. We report all of them below, even if only one gave results which we deemed to be meaningful and we were able to use afterwards.

1. Extracting GWAS traits from OMIM.

We classified the OMIM diseases whose disease-gene associations were obtained through GWAS as *Complex* and the remaining as *Mendelian.* To do this, we developed a method which classifies the OMIM diseases appearing in the EBI GWAS catalogue as *Complex* while the remaining were classified as *Mendelian.*

Considering that the traits in the GWAS catalogue and the OMIM diseases do not have identical names we used an approximate string matching algorithm that produces a similarity score for two given strings. This score (the Levenshtein distance) is based on the number of deletions, insertions and substitutions that are required to match the query strings. We calculated this score for every possible pair GWAS trait - OMIM disease. Our dataset contained 21,529 GWAS traits and 7,812 OMIM diseases, so we obtained a total of 168,184,548 scores. An OMIM diseases was considered to be *Complex* if it was highly similar (similarity > 90%) to a GWAS trait. Unfortunately, this process returned only 60 OMIM diseases being classified as *Complex* which is less than 1% of the total, as well as many diseases classified as *Mendelian* even if many disease genes have already been associated with them.

2. Filtering OMIM based on the Phenotype mapping key

We contacted the staff at OMIM, who recommended us to filter the OMIM database based on the *Phenotype Mapping Key* of the disease. Following their recommendation we built a set of *Complex* diseases with those diseases that had the Phenotype Mapping Key 2, and a set of *Mendelian* diseases with the mapping key 3.

Unfortunately, this process had similar issues as our previous method, as it returned only 63 OMIM diseases being classified as *Complex* which is less than 1% of the total, as well as many diseases (261) classified as *Mendelian* even if many disease genes have already been associated with them.

3. Extracting the multigenic disorders from OMIM

We classified all multigenic diseases (with more than one gene) in OMIM as *Complex* and all monogenic diseases as *Mendelian.* Here we assume, that the multiple disease genes complicate the elucidation of the gene-disease relationship and therefore, multigenic diseases correspond to the set of inherently *Complex* diseases.

In this way we obtained a set of 287 *Complex* diseases and a set of 3,743 *Mendelian* diseases. There is a statistically significant difference (t-test p-value is 10^-350) between the mean number of disease genes associated to the *Complex* diseases (3.61) and to the *Mendelian* diseases (1).

The results of the evaluation on the three datasets, Pfam, PPI and Sequence Similarity, are shown in **Figure 32.** The composite performance of our method is slightly inferior for the set of *Complex* diseases with respect to the *Mendelian* diseases – the overall composite score is 3.08 for *Complex* and 3.13 for *Mendelian*. Interestingly, the method by Park, which uses molecular level information, is the only method that shows the same behaviour; the methods of Robinson and van Driel obtain a better performance on each of the 3 datasets for *Complex* rather than *Mendelian* diseases. Overall, our method is the most stable as it varies the least in performance between the 2 sets of diseases.

Finally, note that in **Figure 32** the coverage of the different methods is determined only for the diseases in the *Complex* and *Mendelian* sets and not for all of OMIM. Nevertheless, the AUC performance of the methods is comparable to those shown in **Figure 1 top** in the main paper, where the methods are evaluated on all diseases in OMIM.

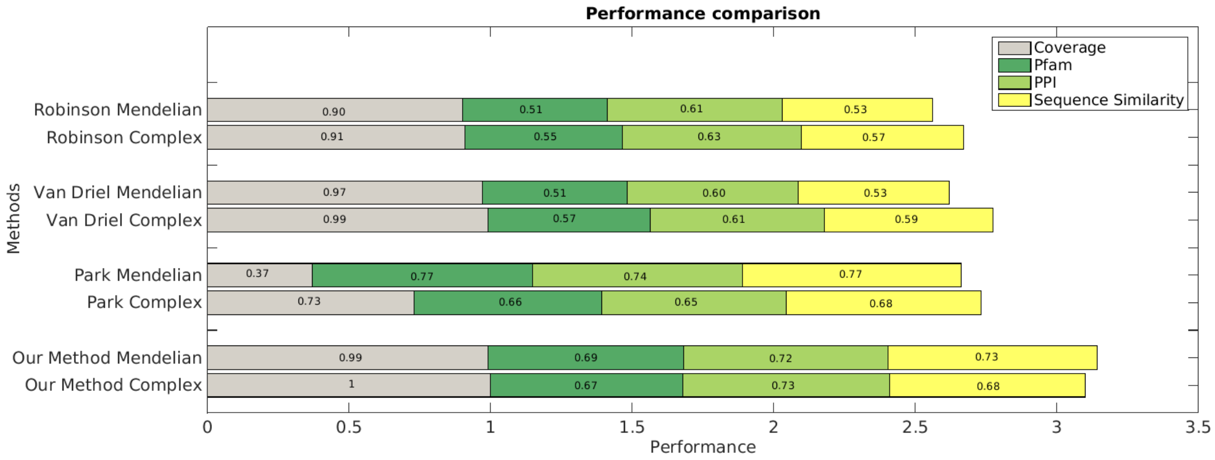

Figure 32 Performance comparison. This figure compares the performance of our method and those by van Driel, Park and Robinson on the Complex and Mendelian sets of diseases. Coverage is defined as the fraction of diseases in the Mendelian and Complex sets for which a similarity can be calculated.

20. The boundary between Goh *et.al* disease classes.

When analysing **Figure 2** in the manuscript it is important to consider that every disease is coloured according to a single class which based on the primary physiological system affected by the disease [2]. Our similarity measure does not determine disease similarity based only on the physiological system affected by the diseases but rather based on the wider aetiology of the disease and also includes risk factors, related drugs and known associations to other diseases. This results in some diseases being placed among diseases of different classes, reflecting the underlying causes of the diseases. This is the case for complex multifactorial diseases such as the *Cardiovascular* diseases.

We have reproduced **Figure 2** from the manuscript in **Figure 33** below and have highlighted diseases classified as *Cardiovascular* that are embedded among other disease classes. MIM 601367 - *Ischemic Stroke* is located in a group of diseases classified as *Metabolic.* There are associations reported between Stroke and metabolic disorders such as MIM 605552 – *AOMS1* [4] and our disease similarity measure scores the pair in the 99th percentile (3.1 similarity). In the same group of *Metabolic* diseases is MIM 608320 - *Coronary artery disease,* whose risk factors include obesity (disease similarity 95th percentile), hypertension (disease similarity 99th percentile), hypercholesterolemia (disease similarity 99th percentile) and diabetes (disease similarity 96th percentile) conditions related to the metabolic system. MIM 255960 - *Myxoma, Intracardiac* is located in the *Cancer* group and has high similarity to

Cancer related disorders such as Carney Complex (99th percentile) and Thyroid Carcinoma (99th percentile). While myxomas are in general benign tumours, they share important hallmarks of cancer.

Contrasting these boundary diseases with the tight group of *Cardiovascular* diseases at the centre of the plot (dashed line) highlights the difference between diseases for which a single class is enough and the previous examples. The conditions represented in this group are intrinsically *Cardiovascular* and are related to mechanical failures of the hearth such as in the cases of 192605 – *Ventricular Tachycardia*, 608567 - *Sick Sinus Syndrome* and 241550 – *Hypoplastic Left Hearth Syndrome* to name a few. These diseases are highly similar with one another and dissimilar to most other diseases in OMIM.

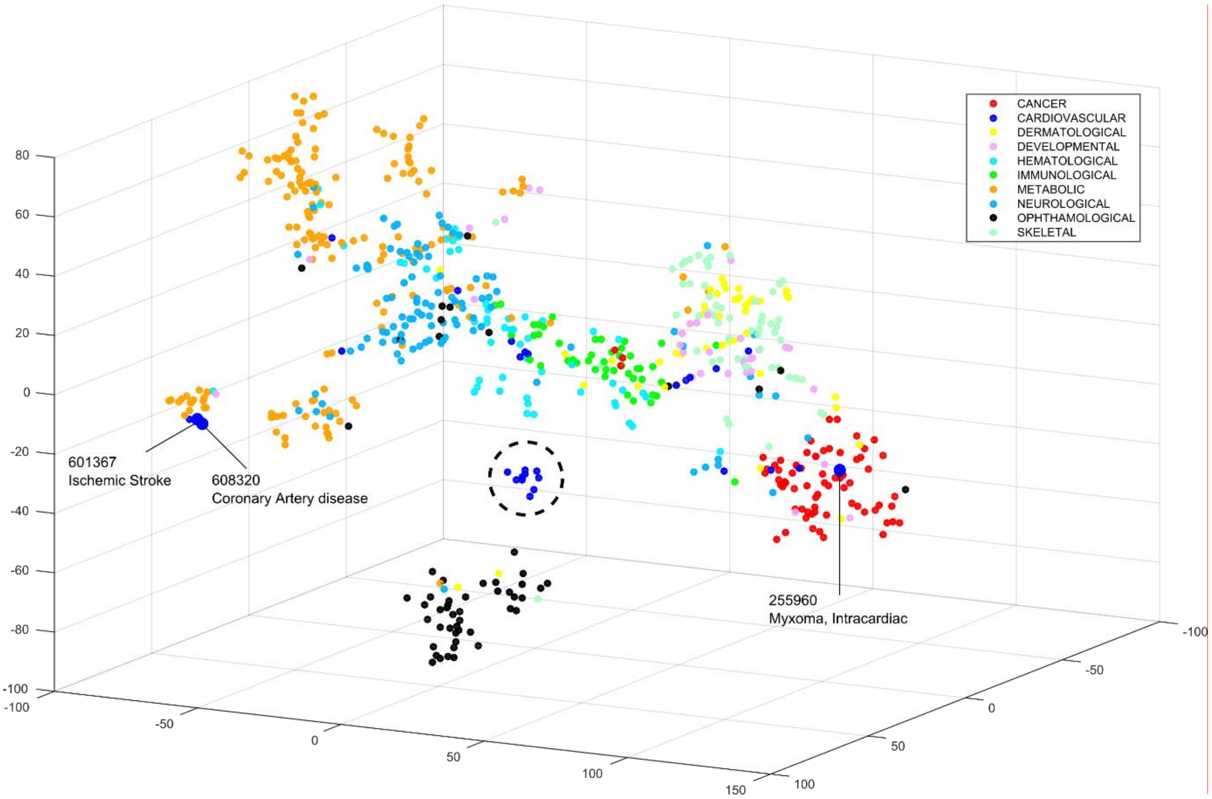

Figure 33 Embedding of OMIM diseases in 3D space. Each point in the plot represents an OMIM disease. The diseases are coloured according to the disease classes in Goh et al. The highlighted diseases correspond to Cardiovascular diseases in the boundary with other classes. The dashed circle shows the tight group of cardiovascular diseases.

21. The impact on the number of genes in a disease pair on its disease similarity score

Diseases with many genes have, on average, higher similarity scores. The higher similarity scores are expected, as diseases with many genes will be more likely to be close to the other diseases in the interactome – informally, we can think of their disease modules as being "larger", and therefore closer.

To show this, we compared the mean similarity of two sets of diseases and all other diseases in OMIM. The first set consists of *multigenic* (strictly more than one gene) and the second set exclusively of *monogenic* (exactly one gene) diseases. The *monogenic* set consists of 3,743 diseases and the *multigenic* set of 287 diseases. The mean similarity between all diseases in the interactome and the diseases in the *monogenic* set is 1.19, compared to the 1.27 between all diseases and the *multigenic* diseases (p-value: 1e-350). We also verify that the *multigenic* diseases are closer to all other diseases in the interactome, compared to the *monogenic* diseases (mean shortest path *multigenic* 4.08, *monogenic* 4.12 p-value: 1e-350).

The number of shared genes between diseases is also reflected in the similarity scores. In **Figure 1 Bottom** in the manuscript we contrast two normalised histograms of disease similarity scores. The yellow histogram in the figure shows the distribution of scores of all pairs of diseases in OMIM, while the green histogram shows the distribution of scores for those diseases in OMIM which share at least one disease gene. The difference between both distributions shown in the figure is statistically significant, and interestingly, 90% of the pairs of diseases which share at least one gene, have similarity scores in the 99th percentile or higher.

We analysed this matter further and counted the number of shared genes in all pairs of diseases. The results are shown in the Table below where we can see that the vast majority of diseases in OMIM do not share any genes.

Number of diseases pairs and the number of genes they share.

Number of shared genes	Number of disease pairs
0	80,830,090
1	6,792

2	22
3	58
4	22
5	17
6	12
7	10
8	3
10	2
11	1
12	1
13	1
15	2
16	1
17	1
18	1
20	1
22	2
35	1

To verify the similarity scores of the diseases that do share genes, we calculated their similarities and represented them in a boxplot shown in **Figure 34.** In this figure, the X-axis correspond to the number of shared genes, and the Y-axis to the distribution of similarity scores, shown as a box-and-whiskers. Each box-and-whiskers diagram represents the median similarity value (indicated by the red line) the upper and lower quartiles (indicated by the box segment below and above the median, respectively) and the maximum and minimum values.

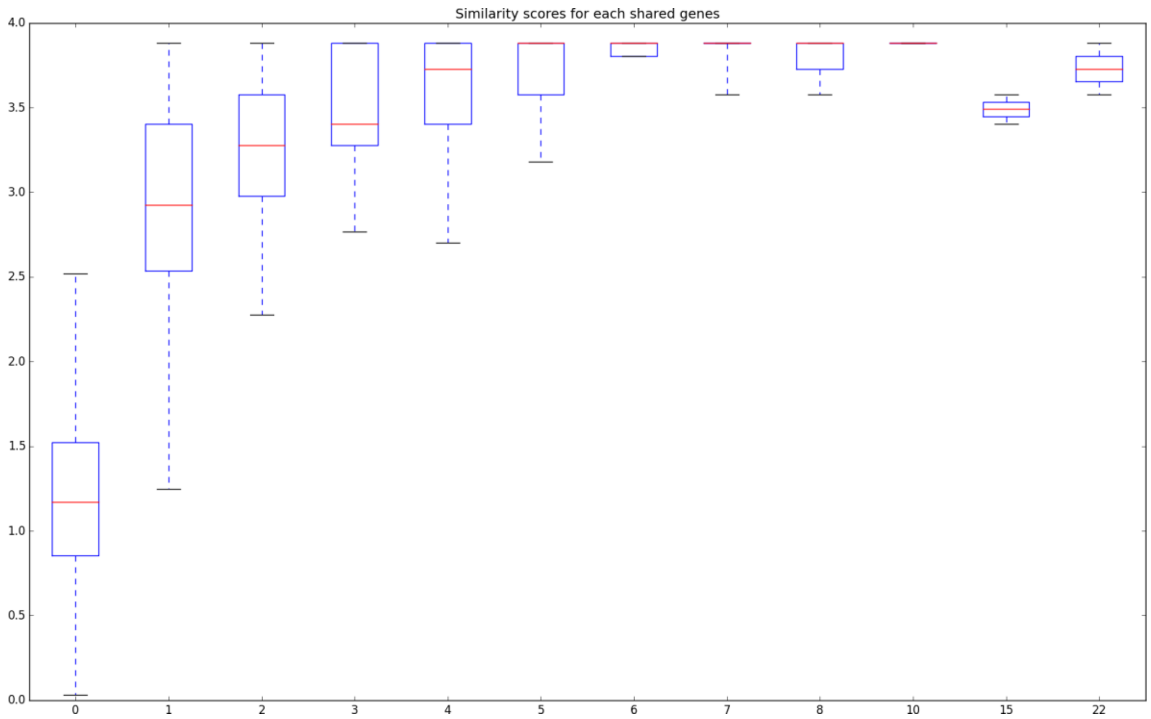

Figure 34 Distribution of similarity scores with respect to the number of shared genes. The plot shows the distribution of similarity scores for pairs of diseases with respect to the number of genes shared by them. The x-axis shows the number of genes shared by the pairs and each corresponding box represents the distribution of scores for those pairs of diseases. The red line in each box represents the median similarity value, the upper portion of each box represents the upper quartile of the distribution and the lower portion the lower quartile.

As we can see, the similarity scores grow the more genes a pair shares. To verify the significance of the difference in the similarity distributions represented in **Figure 34** we performed a pair-wise t-test between the diseases sharing no genes (labelled 0 in the X-axis) an all other diseases, and between the diseases sharing 1 gene and all other pairs. In the Table below we show the p-values of these pairwise t-tests.

	**0**	**1**	**2**	**3**	**4**	**5**	**6**	**7**	**8**	**10**	**15**	**22**
**0**		0.0	0.0	6.7e-261	4.8e-111	5.2e-94	3.7e-72	4e-61	5.8e-19	4.4e-14	1.1e-10	1.0e-12
**1**			3.2e-22	3.2e-13	4.4e-8	2.4e-8	1.3e-7	9.7e-7	0.01	0.01	0.16	0.04

While the significance at the 8 mark drops steeply, we must highlight that for the 8, 10, 15 and 22 mark only 2 disease pairs exist.

22. The disease similarity resource.

We have produced the Disease Similarity Resource (DSR), a database that provides a starting point for transferring knowledge between diseases and possibly the discovery of new disease genes using our measure. The DSR consists of the disease pairs whose similarity scores was in the top 5% (1,552,356 pairs) and their associated disease genes. Each pair constitutes an entry, and contains 5 columns:

1. Disease A
2. Disease B
3. Similarity Score
4. UniProt identifiers of the proteins associated to disease A
5. UniProt identifiers of the proteins associated to disease B

As we have shown in the paper, the high similarity scores between disease pairs indicate that they are likely to be close on the interactome. These highly similar disease pairs are, therefore, suitable candidates for transferring knowledge between them. Thus the DSR provides a starting point for an in-depth analysis into the relationships and aetiology of the diseases, providing the basis for a statistical gene-discovery process. The DSR is freely available in the Downloads section at http://www.paccanarolab.org/disimweb.

23. Evaluating performance of our method on multigenic diseases using ROC curves.

It is important to note that for multigenic diseases the prior probability of having an interacting disease pair (positive) according to one of the relationships (Pfam, PPI and Sequence Similarity) is higher. In fact, when comparing the number of positives in multigenic and monogenic diseases we have: for Pfam 5% vs 1%; for PPI 3% vs 1%; for Sequence Similarity 5% vs 1%. However, the performance of our method does not improve for complex diseases, even if the numbers of positives in the test set is higher; in fact, results are slightly worse (see section 19 of this supplementary material). This is because the area under the ROC curve does not count only the accuracy at predicting positives. By framing the evaluation of our method as a binary classification problem we evaluate, through ROC curves, the capability of the method to predict positives as well as the

capability to predict negatives in the 3 datasets. Thus, when the number of positives in the dataset increases, a method would improve its performance if and only if it were able to produce higher scores only in correspondence to those positives only – an overall mean increase in the scores would not necessarily improve the AUC.

24. Appendix: how to run our disease similarity pipeline

All data is available from www.paccanarolab.org/disease_similarity. The code is released under GPLv3 and is available from https://github.com/pwac092/disim_calculator

Data:

- OMIM data was downloaded on July 21 2014.
- MeSH data corresponds to the 2014 release.

A full browser is available at www.paccanarolab.org/disimweb

Extracting the OMIM data:

We have to manually download the data from omim.org (registration is required) and extract the desired MIM numbers from the catalogue.

Null prefix:

```
$> awk '/*FIELD* TI/{getline;print}' omim.txt | cut -f1 -d " "
| grep "^[0-9]" > phenotypes
```

For all other prefixes (note the changing symbol in the last `grep`)

```
$> awk '/*FIELD* TI/{getline;print}' omim.txt | cut -f1 -d " "
| grep "^+[0-9]" | cut -c2- >> phenotypes
$> awk '/*FIELD* TI/{getline;print}' omim.txt | cut -f1 -d " "
| grep "^#[0-9]" | cut -c2- >> phenotypes
$> awk '/*FIELD* TI/{getline;print}' omim.txt | cut -f1 -d " "
| grep "^%[0-9]" | cut -c2- >> phenotypes
```

Sort the MIM numbers in ascending order

```
$> sort phenotypes -o phenotypes
```

Extracting the referenced publications:

Script: OMIM_query.py

This script will get all the PubMed id's associated to a provided list of MIM numbers. Registration for the API key is required by OMIM. Register for one here http://omim.org/api

Input:

1. The list of MIM numbers produced by the previous step.
2. The name of output file.
3. The path of the configuration file. If no path is provided, `./api_key` will be read.

Output: omim2pubmed maps each OMIM to all of its referenced PubMed identifiers. A tab separated file in the following format is produced

```
OMIM_1 pubmedId . . .
OMIM_2 pubmedId . . .
```

A configuration file is read for the API details using the python `ConfigParser` module. The format is as follows:

```
[APIconfig]
Server = api.europe.omim.org
Key = 5ED0AEDA215A37C589A9AF0E3EAF1F143033E50
```

The API key is provided by OMIM, the one show is for illustration purposes only and as such, is not valid. The server has to be chosen according the one's location.

Additionally, we process the file to extract all the extracted PubMed ids

```
$> cut -f2- omim2pubmed | tr 't' 'n' | sort -n | uniq > unique_pubmedids
```

Keep in mind that there are OMIM records for which no references can be fetched.

Fetching the MeSH terms

Script: PubMed_query.py

This script will fetch the MeSH terms for a given list of PubMed identifiers through API queries to Entrez E-utils.

Input:

1. List of PubMed identifiers for which to fetch the MeSH terms. `unique_pubmedids` from the previous step.
2. Select MajorTopics only
3. Mapping file Double column file, mapping MeSH term names (e.g. Adult) to their unique descriptor identifier (e.g. D000328).
4. Output file name
5. The path of the configuration file. If no path is provided, ./`entrez_config` will be read.

Output

1. A mapping between PubMed identifiers and MeSH terms associated to them. A tab-separated file in the following format is produced

```
PubMedId MeSH MeSH . . .
PubMedId MeSH MeSH . . .
```

PubMed refers to the MeSH descriptors via a unique identifier generated independently. The problem is that there is, to the best of our knowledge, no mapping file that produces the correspondence with MeSH.

However, there is a simple rule to follow. The ids produced by NCBI for MeSH descriptors all start with 68 the ASCII equivalent of which is D. If we transform all the starting 68 by a D we get proper MeSH unique identifiers *e.g.* 68012536 would be translated into D012536

A simple AWK script provides the translation.

```
$> awk '{for(i = 2; i <= NF; i++) gsub("^68","D",$i)}1'
pmids2mesh > pmids2mesh_tr
```

Producing the initial annotations

Script: mim2mesh.py:

This script will map each MIM number to the MeSH terms of its associated PubMed identifiers. It will produce the mapping file also, it will provide a file with all the MIM terms it could not map.

Input:

2. Mapping between OMIM records and the PubMed identifiers of the referenced literature. Tab separated file produced in step 2
3. Mapping between PubMed records and their MeSH terms. Tab separated file produced in step 3

Output:

1. Tab separated file mapping OMIM records to the MeSH terms of its associated publications.

Computing the matrices:

Script: compute_combined_similarity.py

Input:

2. d2014.bin file: This is the plaintext version of all descriptors in MeSH.
3. Annotation file: Initial annotations. In the format:

   `OMIM MeSH MeSH . . .`

   `OMIM MeSH MeSH . . .`

4. Chosen measure. Choices: `resnik, rin, jiang, simui, simGIC`
5. ISM: Optional parameter for improvement of semantic similarity calculations based on [3]. As this was not used for the results provided in the paper, set this to `no`
6. Category subset: Subset of MeSH to use. Options: `all/two/five`. The `five` option was corresponds to our method.
7. Optional filename modifier.

Producing the benchmarks

Now we need to produce the benchmarks, namely the Pfam dataset (Pfam), Protein-Protein interaction dataset (PPI) and Sequence Similarity dataset (SS)

Files are represented as triplets,

`OMIM OMIM 1/0`

Where the last column contains 1 or 0, depending on the physical evidence supporting the similarity of the diseases.

Fetching the sequences

We need to obtain the sequences for the Pfam and SS dataset.

Script: `convert_mimtosp.pl`

This script has no input, it fetches the `mimtosp.txt` file from UniProt and converts it to the `mimtoprot.txt` file. This file maps OMIM records to UniProt identifiers in a format that is easier to process.

Script: `get_sequences.pl`

This script fetches the sequences for the desired proteins.

Input:

1. `Mimtoprot.txt` file

Output:

1. FASTA files of the sequences
2. Unique protein identifiers.

a- Pfam dataset

Sequences have to be fetched for this step. The `pfam_scan.pl` is available from Pfam.

Script: `pfamBenchmark.py`

Two diseases are said to be similar if:

a) Any of their proteins shares at least a Pfam signature.

Input:

b) Result file of the `pfam_scan.pl` script.

c) OMIM to UniProt mapping file

Output:

1. Pfam dataset in triplet form

b- PPI dataset:

Script: `MIM2gene.py`

Two diseases are said to be similar if:

d) Any of the known proteins associated to a disease interacts with the protein of another disease

Input:

2. UniProt ID to Gene Name mapping file.

3. mim2sp file. This file is provided by UniProt and maps OMIM records to their known proteins using UniProt identifiers (http://www.uniprot.org/docs/mimtosp.txt)
4. Protein protein interaction file
5. Valid_OMIM, file of accepted OMIM numbers. In our case the phenotypes file illustrated in step 1.

Output:

6. PPI dataset in triplet form

Different PPI networks can be chosen by defining a vector of columns in the class Interactions in the MIM2gene.py script. After this vector is defined, suffices with appropriately replacing the call to the constructor

```
hprd = Interactions(sys.argv[3], 'columnsHPRD')
```

by replacing 'columnsHRPD' with the vector of columns defined previously.

c- Sequence similarity dataset

Several Perl scripts are required to construct the Sequence Similarity dataset. In the following, they are detailed in the order they should be executed.

Script: `makeblast.pl`

Calculates the smith-waterman alignment of the proteins provided.

Input:

1. FASTA sequence file, *e.g.* produced by `get_sequences.pl`

Output:

1. Smith-Waterman alignment of the proteins

Script: `produce_sequence_similarity.pl`

Produces the Sequence Similarity dataset. Two diseases are positively related when the sequence similarity e-value is lower than 10^-6.

Input:

1. Mimtoprot.txt
2. Smith-Waterman alignment of the proteins.

Output:

1. Sequence Similarity dataset in triplet format

It is important to note that these datasets must be filtered according to the procedure described in section 7 of this Supplementary material.

25. References

1. McKusick-Nathans Institute of Genetic Medicine, J.H.U.B., MD), . *Online Mendelian Inheritance in Man, OMIM®*. 2014.
2. Antonarakis, S.E. and J.S. Beckmann, *Mendelian disorders deserve more attention.* Nat Rev Genet, 2006. **7**(4): p. 277-82.
3. Yang, H., T. Nepusz, and A. Paccanaro, *Improving GO semantic similarity measures by exploring the ontology beneath the terms and modelling uncertainty.* Bioinformatics, 2012. **28**(10): p. 1383-9.
4. Resnik, P., *Semantic similarity in a taxonomy: An information-based measure and its application to problems of ambiguity in natural language.* Journal of Artificial Intelligence Research, 1999. **11**: p. 95-130.
5. Lin, D., *An information-theoretic definition of similarity.* Proceedings of the 15th International Conference on Machine Learning.
6. Jiang, J.J.C., D.W. , *Semantic similarity based on corpus statistics and lexical taxonomy*, in *International Conference Research on Computational Linguistics (ROCLING X)*. 1997: Taiwan. p. 9008–9022.
7. Pesquita, C., et al., *Metrics for GO based protein semantic similarity: a systematic evaluation.* BMC Bioinformatics, 2008. **9 Suppl 5**: p. S4.
8. van Driel, M.A., et al., *A text-mining analysis of the human phenome.* Eur J Hum Genet, 2006. **14**(5): p. 535-42.
9. Park, S., et al., *Protein localization as a principal feature of the etiology and comorbidity of genetic diseases.* Molecular Systems Biology, 2011. **7**.
10. Robinson, P.N., et al., *The Human Phenotype Ontology: a tool for annotating and analyzing human hereditary disease.* Am J Hum Genet, 2008. **83**(5): p. 610-5.
11. Zhou, X., et al., *Human symptoms-disease network.* Nat Commun, 2014. **5**: p. 4212.
12. Goh, K.I., et al., *The human disease network.* Proc Natl Acad Sci U S A, 2007. **104**(21): p. 8685-90.
13. Kibbe, W.A., et al., *Disease Ontology 2015 update: an expanded and updated database of human diseases for linking biomedical knowledge through disease data.* Nucleic Acids Res, 2014.
14. Ashburner, M., et al., *Gene ontology: tool for the unification of biology. The Gene Ontology Consortium.* Nat Genet, 2000. **25**(1): p. 25-9.
